# Anthropogenic emission is the main contributor to the rise of atmospheric methane during 1993–2017

**DOI:** 10.1093/nsr/nwab200

**Published:** 2021-11-11

**Authors:** Zhen Zhang, Benjamin Poulter, Sara Knox, Ann Stavert, Gavin McNicol, Etienne Fluet-Chouinard, Aryeh Feinberg, Yuanhong Zhao, Philippe Bousquet, Josep G Canadell, Anita Ganesan, Gustaf Hugelius, George Hurtt, Robert B Jackson, Prabir K Patra, Marielle Saunois, Lena Höglund-Isaksson, Chunlin Huang, Abhishek Chatterjee, Xin Li

**Affiliations:** Department of Geographical Sciences, University of Maryland, College Park, MD 20742, USA; Biospheric Sciences Laboratory, NASA Goddard Space Flight Center, Greenbelt, MD 20771, USA; Department of Geography, University of British Columbia, Vancouver V6T 1Z2, Canada; Global Carbon Project, CSIRO Oceans and Atmosphere, Canberra, ACT 2601, Australia; Department of Earth and Environmental Sciences, University of Illinois Chicago, Chicago, IL 60607, USA; Department of Earth System Science, Stanford University, Stanford, CA 94305, USA; Institute for Data, Systems and Society, Massachusetts Institute of Technology, Cambridge, MA 02139, USA; College of Oceanic and Atmospheric Sciences, Ocean University of China, Qingdao 266000, China; Laboratoire des Sciences du Climat et de l’Environment, LSCE-IPSL (CEA-CNRS-UVSQ), Université Paris-Saclay, Gif-sur-Yvette 91191, France; Global Carbon Project, CSIRO Oceans and Atmosphere, Canberra, ACT 2601, Australia; School of Geographical Sciences, University of Bristol, Bristol BS8 1RL, UK; Department of Physical Geography and Bolin Centre for Climate Research, Stockholm University, Stockholm SE-106 91, Sweden; Department of Geographical Sciences, University of Maryland, College Park, MD 20742, USA; Department of Earth System Science, Stanford University, Stanford, CA 94305, USA; Woods Institute for the Environment and Precourt Institute for Energy, Stanford University, Stanford, CA 94305, USA; Research Institute for Global Change, JAMSTEC, Yokohama 236-0001, Japan; Laboratoire des Sciences du Climat et de l’Environment, LSCE-IPSL (CEA-CNRS-UVSQ), Université Paris-Saclay, Gif-sur-Yvette 91191, France; International Institute for Applied Systems Analysis (IIASA), Laxenburg A-2361, Austria; Northwest Institute of Eco-Environment and Resources, Chinese Academy of Sciences, Lanzhou 730000, China; Global Modeling and Assimilation Office, NASA Goddard Space Flight Center, Greenbelt, MD 20771, USA; Universities Space Research Association, Columbia, MD 21046, USA; Institute of Tibetan Plateau Research, Chinese Academy of Sciences, Beijing 100101, China

**Keywords:** greenhouse gas, carbon cycle, climate mitigation, wetland, methane isotope

## Abstract

Atmospheric methane (CH_4_) concentrations have shown a puzzling resumption in growth since 2007 following a period of stabilization from 2000 to 2006. Multiple hypotheses have been proposed to explain the temporal variations in CH_4_ growth, and attribute the rise of atmospheric CH_4_ either to increases in emissions from fossil fuel activities, agriculture and natural wetlands, or to a decrease in the atmospheric chemical sink. Here, we use a comprehensive ensemble of CH_4_ source estimates and isotopic δ^13^C-CH_4_ source signature data to show that the resumption of CH_4_ growth is most likely due to increased anthropogenic emissions. Our emission scenarios that have the fewest biases with respect to isotopic composition suggest that the agriculture, landfill and waste sectors were responsible for 53 ± 13% of the renewed growth over the period 2007–2017 compared to 2000–2006; industrial fossil fuel sources explained an additional 34 ± 24%, and wetland sources contributed the least at 13 ± 9%. The hypothesis that a large increase in emissions from natural wetlands drove the decrease in atmospheric δ^13^C-CH_4_ values cannot be reconciled with current process-based wetland CH_4_ models. This finding suggests the need for increased wetland measurements to better understand the contemporary and future role of wetlands in the rise of atmospheric methane and climate feedback. Our findings highlight the predominant role of anthropogenic activities in driving the growth of atmospheric CH_4_ concentrations.

## Teaser

Nature or humans? Scenarios for constraining why atmospheric methane continues to rise point to agriculture and fossil fuels.

## INTRODUCTION

Stabilizing atmospheric methane (CH_4_) emissions from anthropogenic activities is a critical component of climate change mitigation [1]. The atmospheric CH_4_ concentration has increased ∼2.5-fold from 731 ppb (parts per billion) in 1750 (pre-industrial reference year [2]) to 1890 ppb in 2020 [3]. Meanwhile, over the past century, atmospheric δ^13^C-CH_4_ values increased from ∼−49.0‰ in 1912 to −47.2‰ in 2007 due to increasing emissions of isotopically ^13^C-enriched (i.e. isotopically heavy) fossil fuels [4]. Despite the importance of understanding the temporal changes in atmospheric CH_4_, the drivers of changes in the growth rate of atmospheric CH_4_ over recent decades remain poorly understood [5]. The increase in atmospheric CH_4_ slowed in the early 1990s and was followed by a so-called stabilization period during 2000–2006 [6]. Since 2007, global atmospheric CH_4_ concentrations have begun to rise again, accompanied by a decline in δ^13^C-CH_4_ values from −47.2‰ in 2007 to −47.4‰ in 2017 [3]. The cause of this change has been studied recently using atmospheric inversion models [7–10], atmospheric box models [11–15] and emission inventories [16]. These studies have arrived at divergent and even conflicting conclusions [17], citing increasing emissions of CH_4_ from fossil fuels, agriculture, wetlands and/or decreased hydroxyl radicals (OH) as main drivers due to different measurements, methodologies and time periods considered (see Materials and Methods). Such discrepancies highlight the need to reconcile our understanding of the drivers of growth in atmospheric CH_4_ in order to design mitigation policies [18].

Sources of the global CH_4_ budget are mainly determined by three broadly defined groups: (i) thermogenic sources from industrial fossil fuel (e.g. coal, oil and natural gas; IFF_CH4_) and geological sources (GEO_CH4_); (ii) biogenic sources from livestock, rice agriculture, landfills and waste (AGW_CH4_), and natural wetlands (WET_CH4_); and (iii) pyrogenic sources from wildfires and biomass burning (BB_CH4_). The primary sink for CH_4_ is reactions with tropospheric OH, soil microbial uptake and a small contribution from tropospheric chlorine reactions, which affect the isotopic compositions. The shift of the trend in atmospheric δ^13^C-CH_4_ values towards more ^13^C-depleted (i.e. isotopically light) compositions suggests a higher dominance of isotopically light biogenic emissions in the global CH_4_ budget [11,19]. This hypothesis has been supported by recent process-based and inversion modeling, which points to either a systematic underestimation of AGW_CH4_ [20] or a large increase in WET_CH4_ [8,10,21,22]. In contrast, there are large differences in the rate of change across inventory-based estimates of industrial fossil fuel source activity IFF_CH4_ [23,24], as well as substantial underestimates in some regions and overestimates in other regions [12,25,26]. Globally, studies suggest that BB_CH4_ has been declining, with fire CH_4_ emissions associated with an isotopically enriched signature, thus providing room in the isotopic budget for an increase in fossil fuel sources [13]. GEO_CH4_, which is often co-located with the fossil fuel industry, is suggested to be largely overestimated by recent studies [27,28], indicating a potentially larger role of IFF_CH4_ in affecting the global CH_4_ budget given its underestimated share of the total CH_4_ source [12,29].

OH oxidation in the troposphere is the main CH_4_ sink, and reactions with chlorine (Cl), stratospheric sinks and soil removal are small-magnitude sinks. Substantial difficulties remain in quantifying CH_4_ sinks, especially the main chemical sink for CH_4_, tropospheric OH [5]. OH plays a significant but ambiguous role in driving the observed atmospheric trend; it is difficult to estimate due to its complicated chemistry, i.e. non-linear chemical feedback and short lifetime [30,31]. For example, estimates of interannual variability (IAV) in global mean OH are significantly higher in the empirical box-model estimates that use CO and methylchloroform (MCF) constraints [32] than estimates based on chemical transport models (Fig. S1). Although there are debates on the potential biases in the box-model-based OH due to ignorance of complex spatial heterogeneity in OH and transport [33,34], the uncertainties in OH trends and variability are likely large enough to explain any potential CH_4_ growth scenarios [14,15,35]. In addition, the trends in OH exert isotopic leverage on atmospheric δ^13^C-CH_4_ values via the kinetic fractionation effect, such that increasing OH increases the atmospheric δ^13^C-CH_4_ value by OH reacting with more ^12^CH_4_. Therefore, it is of interest to investigate hypotheses regarding CH_4_ sources with atmospheric δ^13^C-CH_4_ observations while assuming that these sources can reproduce the atmospheric records with varying OH.

A thorough investigation of these hypotheses in a clearly defined framework is essential to help resolve the unexplained change in the growth rate of atmospheric CH_4_. Here, we use an isotopic mass balance approach to attribute drivers of the growth rate of atmospheric CH_4_ using a large ensemble of scenarios to represent different combinations of emission hypotheses (denoted emission scenarios, see Materials and Methods) from a comprehensive set of updated bottom-up estimates representing anthropogenic emission inventories and spatially explicit signatures for major CH_4_ sources. Each emission scenario is composed of a time series of sectoral CH_4_ fluxes and their hemispheric emission-weighted δ^13^C-CH_4_ values. A globally representative database and spatially resolved distributions of δ^13^C-CH_4_ values for the major CH_4_ sources [36–38] were used to evaluate the temporal and regional variability in observed δ^13^C-CH_4_ values. Monte Carlo techniques were applied to explore the uncertainty in δ^13^C-CH_4_ estimates with full consideration of the spatial heterogeneity in CH_4_ sources and their δ^13^C-CH_4_ signatures. Scenario-specific parameters for the time series of the CH_4_ removal rate driven by OH variations and run-specific ^13^CH_4_ fractionation factors were derived by inverting an atmospheric two-box model (see Methods and Supplementary Data). We then evaluated the emission scenarios against observed δ^13^C-CH_4_ values for 1993–2017 by running the two-box model in the forward mode. To test the hypothesis of a large increase from wetland CH_4_ emissions, the idealized wetland scenarios (i.e. without process-based constraint) were then calculated to reproduce the temporal pattern of δ^13^C-CH_4._ The comparison against atmospheric isotopic observations allowed us to select the most likely set of emission scenarios, which are defined as the first percentile cut-off of the lowest mean squared difference (MSD) in simulated δ^13^C-CH_4_ values.

## RESULTS AND DISCUSSION

### Temporal variations in the atmospheric CH_4_ concentration and its **δ**^13^C-CH_4_ value

**Figure 1.**
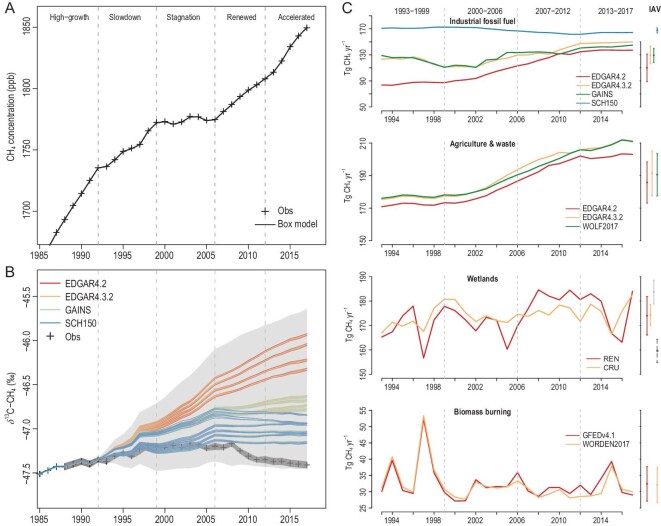
Simulated global atmospheric CH_4_ concentration, δ^13^C-CH_4_ values and bottom-up estimates of major CH_4_ sources. (A) Simulated atmospheric CH_4_ concentration (black solid line) from all emission scenarios exactly reproducing the observed CH_4_ records (cross dots). The ensemble simulations of the box model were run in forward mode with prescribed δ^13^C-CH_4_ variations from emission scenarios using OH time series derived from inverse mode (Fig. S1). (B) Simulated ensemble mean δ^13^C-CH_4_ values (colored solid lines) for emission scenarios grouped by industrial fossil fuel inventories (EDGARv4.2, EDGARv4.3.2, GAINS and SCH150) in comparison to the observed global mean δ^13^C-CH_4_ (cross). The uncertainty range of ensemble simulations (*n* = 96 000) and 1-σ uncertainty of the observations are shown as light gray areas and dark gray areas, respectively. (C) Time series of annual total emissions for major sources. The interannual variability (1-σ) in CH_4_ for each individual bottom-up estimate is shown at the right of the plot. Note that for the wetland category, the dashed bars represent two independent estimates from a bottom-up ensemble of wetland models [40] for 2000–2012 (light gray) and WetCHARTs [41] (dark gray) for 2001–2015.

The ensemble simulations for the 96 emission scenarios (4 IFF_CH4_ × 3 AGW_CH4_ × 2 WET_CH4_ × 2 BB_CH4_ × 2 GEO_CH4_) reproduced the observed atmospheric CH_4_ concentration (Fig. 1A) using the corresponding optimized time series of OH derived from running the box model in inverse mode (Fig. S1). We accounted for the uncertainty in source signatures of δ^13^C-CH_4_ by resampling 1000 sets of δ^13^C-CH_4_ signature time series for each emission scenario (*n* = 96 × 1000), which resulted in a wide range of modeled atmospheric δ^13^C-CH_4_ values with some closely reproducing the observations. However, most emission scenarios tended to generate more enriched δ^13^C-CH_4_ trends for 1993–2017, suggesting that existing bottom-up inventories overestimate the increase in IFF_CH4_ during the study period, especially during slowdown and stagnation periods (Fig. 1B). Furthermore, to balance the rise in CH_4_ sources, the increases in OH levels also led to positive trends in atmospheric δ^13^C-CH_4_ values during these two periods (Fig. S1). Note that the large increase in coal-related emissions since 2003 has increased the δ^13^C-CH_4_ values of the sources, which contribute to the divergence between the model and observations. The timing of this divergence is consistent with a rapid increase in methane emissions from China (mainly coal emissions) as reported by the inventories [23] and inversions [39]. For most of the emission scenarios, the estimated temporal variations in OH for 1993–2017 fall within the 1-σ range of the Bayesian inversion from Ref. [14] and are in good agreement with Ref. [35] for the post-2000 period. The EDGARv4.2-based emission scenarios have the largest mismatch with the inversion-based OH anomaly (relative to a global mean concentration of 1e^6^ molecules (molec)/cm^3^), confirming the known higher bias in EDGARv4.2 than in other inventories (Fig. S2).

The time series of CH_4_ sources for 1993–2017 (Fig. 1C) suggests that decadal-scale variations in atmospheric CH_4_ are dominated by anthropogenic emissions from both agricultural and fossil fuel activities. However, there is high uncertainty across IFF_CH4_ inventories, with a sizable (>40 Tg CH_4_ yr^–1^) difference in magnitude and a large difference in temporal trends between inventories (i.e. EDGARv4.2, EDGARv4.3.2 and GAINS) and an atmospheric-observation-constrained approach (i.e. SCH150, which hypothesizes that IFF_CH4_ is underestimated but does not increase over time). The temporal variation in AGW_CH4_ exhibits a lower discrepancy than IFF_CH4_ in the inventories, whereas WET_CH4_ and BB_CH4_ are more constrained. The IAV and magnitude of our estimates for WET_CH4_, calculated using the process-based model LPJ-wsl, are comparable to the ensemble mean of multiple wetland models [40] and a global wetland CH_4_ emission model ensemble for use in atmospheric chemical transport models (WetCHARTs) [41]. The wetland CH_4_ estimates derived from driving the wetland model with a ground-measurement-based meteorological dataset from the Climate Research Unit (CRU) yield a small increase (<1 Tg CH_4_ yr^–1^), whereas the same model with climate reanalysis (REN) has an ∼7.3 Tg CH_4_ step increase from tropical wetlands between 2000–2006 and 2007–2017 [22].

### Evaluations of proposed CH_4_ hypotheses using emission scenarios

**Figure 2.**
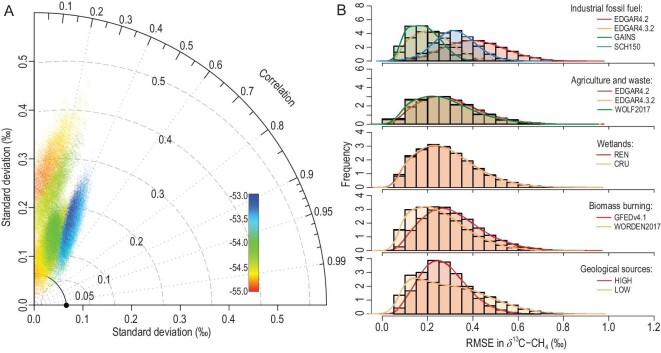
Performance of emission scenarios in simulating atmospheric δ^13^C-CH_4_. (A) Taylor diagram illustrating the similarity between individual time series from the 96 000 simulations of different emission scenarios (colored dots) to the observed atmospheric δ^13^C-CH_4_ for 1993–2017. The black solid dot refers to the observed global average δ^13^C-CH_4_ for 1993–2017. Each dot symbol indicates the correlation value (angle), the standard deviation (SD, radial distance to the origin point) and the root mean square error (RMSE, distance to the black solid dot), with different colors representing the mean global average δ^13^C-CH_4_ value of the source. (B) Histograms of RMSE between simulated δ^13^C-CH_4_ values and observations grouped by bottom-up estimates with different colors for major CH_4_ source categories. The solid lines represent the fitted density distribution after spline smoothing.

Figure 2A shows the distribution of residual bias in the individual box model simulations in terms of how they reproduce the observed δ^13^C-CH_4_ values. In the Taylor diagram [42] the global average δ^13^C-CH_4_ values of the sources before fractionation by chemical sinks range from −53‰ to −55‰ over 1993–2017, and a correlation coefficient lower than 0.6 is obtained for all of the simulations for 1993–2017. The low agreement suggests that the biases in the inventories and the wetland models contribute to the discrepancies in reproducing the δ^13^C-CH_4_, which is likely due to the overestimated increase in inventories, especially the coal emission that has a relatively heavy isotopic signature, as found by previous atmospheric inversion studies [16]. In addition, some of the simulations can reproduce similar IAV in atmospheric δ^13^C-CH_4_ values with root mean square errors (RMSEs) from 0.05‰ to 0.5‰, but ∼85% of simulations tend to produce higher IAV than observed. Although the global average δ^13^C-CH_4_ value was regarded as observational ‘truth’, this reference has an uncertainty of 0.04‰, attributed to variability in measurements across all the stations and uncertainty from scale conversions between networks [43].

The density distributions of RMSEs grouped by different bottom-up CH_4_ estimates show the divergent performance of emission scenarios in reproducing observed atmospheric δ^13^C-CH_4_ values (Fig. 2B). Among the four IFF_CH4_ inventories, 80% of the simulations using EDGARv4.2 generated more positive trends of δ^13^C-CH_4_ values than the other three inventories, with no EDGARv4.2-based runs located in the most likely set of emission scenarios (Fig. S3). This result corroborates previous studies that also suggest that EDGARv4.2 tends to overestimate fossil fuel growth [44]. The more recent EDGARv4.3.2 has been improved, with 95% of the simulations located within the RMSE range from 0.1‰ to 0.3‰, mainly due to improved emission factors and revised statistics of CH_4_ sectors [23]. SCH150 produces lower agreement than EDGARv4.3.2 partly due to the low IAV of SCH150, as SCH150 focuses on the long-term trends in IFF_CH4_. The GAINS inventories generated better performance: 78% of the simulations in the first percentile of MSD are based on GAINS IFF_CH4_. Note that this does not rule out the IFF_CH4_ scenarios that have flat or insignificant trends (e.g. SCH150), as ^13^C-enriched BB_CH4_ estimates in this study show declining trends over recent years, which would allow for compensation by increasing emissions from IFF_CH4_ to meet the decreasing atmospheric δ^13^C-CH_4_ values. Generally, IFF_CH4_ has a more pronounced impact in determining the past trends in δ^13^C-CH_4_ changes than the other major CH_4_ sources.

The contribution of combined agriculture, landfills and waste included in AGW_CH4_, which together represent 50%–62% of all anthropogenic sources, again reveals a higher bias of EDGARv4.2-based simulations compared to the other two inventories (Fig. 2B). Agricultural emissions dominate AGW_CH4_, with an average contribution of 77.5% to the total AGW_CH4_ over the study period. The lower MSD scores using EDGARv4.3.2 and WOLF2017 emission scenarios show improved reconciliation of estimates for the AGW_CH4_ source relative to the isotopic budget. This result supports the hypothesis that the global livestock estimates based on the 2006 IPCC Tier 1 guidelines underestimate livestock CH_4_ emissions at the national or state level [45], which is potentially attributable to outdated information used to develop the emission factors. However, there is no clear signal to distinguish whether EDGARv4.3.2 or WOLF2017 has a lower a priori bias, suggesting the need for further regional and global assessments by spatially explicit 4-D atmospheric models.

Our calculations suggest that, in contrast to anthropogenic sources, wetland CH_4_ emissions play a limited role in reproducing the decadal trend in atmospheric δ^13^C-CH_4_ (Figs 1 and 2B). Both REN and CRU demonstrate that wetland CH_4_ emissions appear to have contributed little to the renewed growth in atmospheric CH_4_. However, wetland emissions help explain the IAV in the atmospheric CH_4_ growth rate via its pulsed responses to climate dynamics, such as the El Niño-Southern Oscillation [46]. The latitudinal gradient of the growth rate for CH_4_ sources (Fig. S4) suggests that WET_CH4_ in the tropics has an important impact on the IAV of the CH_4_ growth rate, albeit the current limited understanding of WET_CH4_ is due to a significant deficiency in WET_CH4_ measurements in the tropics, especially for Africa [21].

The density distribution of RMSE grouped by BB_CH4_ and GEO_CH4_ (Fig. 2B) suggests that the recent hypotheses regarding a larger decrease [13] in BB_CH4_ and overestimated contemporary GEO_CH4_ [27,28] have a good agreement with the isotopic budget. The lower RMSE of Worden (2017)-based scenarios supports the hypothesis of a decreasing trend in BB_CH4_ during the post-2007 period, as suggested by inversion modeling based on satellite measurements of carbon monoxide [47]. The low GEO_CH4_ scenarios, which assume a geological source of 15 Tg CH_4_ yr^–1^ with upward-revised IFF_CH4_ (see Methods and Supplementary Data), yield lower RMSEs than the conventional high-GEO_CH4_ scenarios in which GEO_CH4_ was set to 52 Tg CH_4_ yr^–1^. These findings support the hypothesis that the current bottom-up estimates of anthropogenic fossil fuel CH_4_ emissions are underestimated and that geological emissions are overestimated.

### Changes in the trends of **δ**^13^C-CH_4_ source signatures

**Figure 3.**
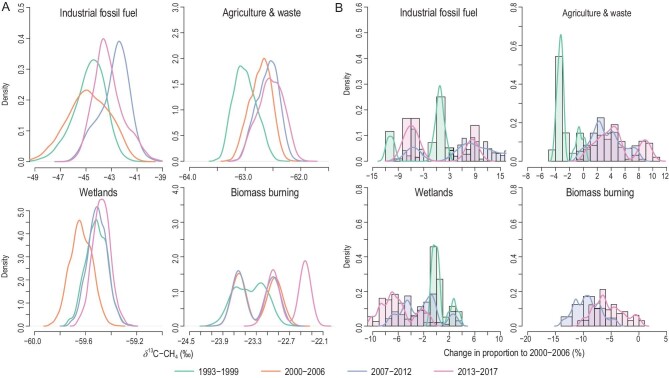
Changes in δ^13^C-CH_4_ values and in contribution of ^13^CH_4_ mass to the annual total of ^13^CH_4_ mass for major source categories during 1993–2017. (A) Density function of the mean emission-weighted δ^13^C-CH_4_ value of the sources for four time periods from Monte Carlo accounting (*n* = 96 000): decreased atmospheric growth during 1993–1999, relative stabilization during 2000–2006, renewed growth during 2007–2012 and accelerated growth during 2013–2017. While the emission-weighted δ^13^C-CH_4_ signatures may change over time, it is the combination of these signatures with their respective emission amounts that determines the atmospheric isotopic trend. (B) Density distribution of changes in the contribution of ^13^CH_4_ mass from major CH_4_ source categories to annual global ^13^CH_4_ mass. The contribution of ^13^CH_4_ mass is calculated as the average percentage change of the ratio of average annual total ^13^CH_4_ mass in the source during 1993–1999, 2007–2012 and 2013–2017 relative to the CH_4_ plateau period 2000–2006.

A change in source signature (Fig. 3A) suggests varying global-emission-weighted average sources driven by the change in spatiotemporal distribution of CH_4_ source estimates for the four major CH_4_ categories. When considering spatial heterogeneity in the source signature, the globally representative δ^13^C-CH_4_ values tend to suggest a larger variation than previous assumptions that use globally uniform values [7,13]. The IFF_CH4_ signature varies between time periods from a median of −44.9‰ during 2000–2006 to a median of −42.7‰ during 2013–2017, suggesting high variability in the δ^13^C-CH_4_ values of anthropogenic CH_4_ sources. The AGW_CH4_ signature slightly increased from a median value of −62.5‰ to −62.3‰ from 2000–2006 to post-2007. Note that the effect of the decreasing trend of atmospheric δ^13^C-CO_2_ values on the C_3_–C_4_ diet composition of domestic ruminants in recent decades was not taken into account in this study; consideration of this factor would yield a slight decrease in the AGW_CH4_ signature [48]. Wetland δ^13^C-CH_4_ values increased slightly from −59.7‰ to −59.5‰ from the stabilization period to the renewed-growth period, mainly attributable to increased tropical wetland CH_4_ emissions since 2007. Tropical wetlands tend to have a more enriched signature (mean −56.7‰) than northern high-latitude peatland-based wetlands (mean −67.8‰) (Fig. S5), as supported by a few site-level measurements [36,38,49]. The possible signature enrichment from wetlands is another line of evidence for a weak wetland CH_4_ emission response [22,40], while there is no evidence of a significant change in wetland CH_4_ from high latitudes in either model [16,44] or by direct atmospheric measurement [50], where the rise of WET_CH4_ may possibly be counteracted by increased soil uptake [51]. However, it is difficult to distinguish CH_4_ from wetlands and livestock, as the signatures of the two sectors are similar and the spatial distributions are possibly co-located [3], suggesting a critical need for more measurements to provide better constraints on δ^13^C-CH_4_ values in the tropics.

The change in the δ^13^C-CH_4_ contribution from individual sources does not necessarily imply the same trend in the global average signature. Theoretically, even if the tropical wetland signature becomes more positive, the increased proportion of wetland-contributed ^13^CH_4_ mass to the total ^13^CH_4_ mass can still result in a shift towards a more negative global signal, as the biogenic signature is considerably lighter than the global atmospheric δ^13^C-CH_4_ value [36] (∼−53.6‰) before fractionation. This is the case in some paleoclimate studies [52] where tropical wetlands and other natural sources (e.g. biomass burning) dominated the annual CH_4_ budget. However, the role of human activities has become dominant in the annual CH_4_ and isotope budgets since AD 1750, and the relative importance of wetlands has lessened. Figure 3B also shows the probability distribution of the relative contribution of ^13^CH_4_ mass to the annual total ^13^CH_4_ mass in the source, from 2000–2006 to the post-2007 period. IFF_CH4_ exhibits either an increased contribution of 5%–8% based on EDGARv4.2 or a decreased contribution of 6%–9% relative to 2000–2006 based on SCH150 or GAINS. In contrast to IFF_CH4_, AGW_CH4_ shows a significantly increasing contribution to the isotope budget from 2000–2006 to the post-2007 period, with a positive trend of 5%–7% relative to 1993–1999. This pattern can be explained by the substantial increase in AGW_CH4_ production since the 21st century. The contribution of BB_CH4_ to the ^13^CH_4_ mass was 20%–25% lower in 2007–2017 than in 1993–1999 and 2000–2006, mainly due to reduced biomass burning, as suggested by the inversion model based on satellite retrievals [13] and by inventories [47].

### Idealized wetland emission scenarios that reproduce the decrease in atmospheric **δ**^13^C-CH_4_ values

**Figure 4.**
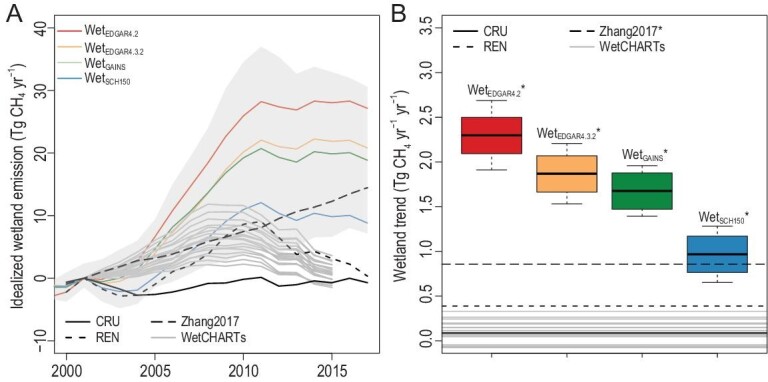
Idealized wetland emissions that reproduce the observations of atmospheric CH_4_ and δ^13^C-CH_4_ for 2000–2017. (A) Time series of anomalies of idealized WET_CH4_ with a 7-year moving window (colored lines: ensemble mean grouped by IFF_CH4_) in comparison to the two emission scenarios (CRU and REN) applied in this study and the estimates from Ref. [53] (denoted Zhang2017) and WetCHARTs [41]. All estimates are anomalies relative to 2001. The min/max range of idealized wetland emissions is shown as the gray area. (B) Trend of wetland emissions for 2000–2017 computed using linear regression. Significant trends at the 95% confidence level are denoted with ‘*’.

Beyond our wetland-model ensemble, we created scenarios to investigate the possible involvement of rising WET_CH4_ in the decrease of atmospheric δ^13^C-CH_4_ values. To do so, we performed a sensitivity test by running the box model in inverse mode for each individual run to calculate idealized WET_CH4_ given the other sources, varying OH concentration, atmospheric CH_4_ and δ^13^C-CH_4_ observations, and the isotopic signatures of the sources, which thus linearizes the problem. The results suggest that the magnitude of increase in idealized WET_CH4_ largely depends on the hypothesis of IFF sources, where greater wetland increases are required to compensate for the large increase in the IFF_CH4_ scenarios (Fig. 4A). Note that all the idealized WET_CH4_ scenarios are higher than the two WET_CH4_ scenarios in this study (i.e. CRU and REN) or WetCHARTs, a wetland CH_4_ product that is based on satellite-derived surface water extent and precipitation reanalysis and an ensemble of ecosystem respiration estimates. One process-based WET_CH4_ that overlaps the increases in idealized wetland scenarios is the ensemble mean of LPJ-wsl simulations for a future projection under the climate scenario RCP8.5 [53] (denoted Zhang2017). RCP8.5 is considered the upper bound of wetland CH_4_ feedback to rising temperature in LPJ-wsl because the strong and steady increase in temperature in RCP8.5 is higher than that determined from actual observations. Note that this scenario would occur only in combination with the hypothesis that IFF_CH4_ has had no significant trends in recent years.

The comparison of 2000–2017 trends in WET_CH4_ (Fig. 4B) suggests that to reproduce the magnitude of the observed decrease in atmospheric δ^13^C-CH_4_ values, the required emission increase from natural wetlands would need to be much higher than the current estimates from process-based wetland models. The trend of CRU is consistent with the ensemble estimate of global wetland model simulations [5,40], while that of REN is at the higher end of the trends that consider the potential inundation increase due to enhanced tropical precipitation [22]. Note that the range of idealized increases in WET_CH4_ is in line with two recent inversion studies [8,10] based on GOSAT CH_4_ measurements, which suggests a positive wetland trend of 2–3 Tg CH_4_ yr^–1^ yr^–1^ for 2010–2018. However, to produce such a significant trend, the Q10 parameter (temperature sensitivity of CH_4_ emissions) in the wetland models would need to be much higher than the range of 2–3 from LPJ-wsl and WetCHARTs or the measurement-based average of 2.57 from FLUXNET-CH4 [54]. In addition, a recent multi-model ensemble inversion study [55] suggests that the observation-constrained wetland CH_4_ feedback to rising temperature is lower than that of Zhang 2017. Despite this, there are considerable uncertainties in modeled WET_CH4_ due to scarcity of measurements for the tropics [40]. We conclude that the hypothesis that a large increase in natural wetlands drives the decrease in atmospheric δ^13^C-CH_4_ values cannot be reconciled with process-based wetland CH_4_ models.

### Attributions of the CH_4_ rise based on the most likely scenarios

Our Monte Carlo estimation (Table 1) suggests that the largest uncertainties in global representative source δ^13^C-CH_4_ values are in industrial fossil fuel activities, providing clues for future studies. Our estimated global representative values for total CH_4_ source signatures are within the uncertainty of recently compiled databases [12,36] but are lower than the value used in previous inverse studies (see Fig. S6 for references). Among the major CH_4_ emission sectors, the global average emission-weighted δ^13^C-CH_4_ signature for coal has the highest IAV, which is mainly due to the large deviation in country-level data in coal emissions [39] and the heterogeneous distribution of different coal ranks [56]. Note that the low-rank coals tend to produce isotopically lighter CH_4_ [36] with a potentially biogenic origin [57], indicating that the proportion of consumption of different coal types may have a significant impact on atmospheric δ^13^C-CH_4_ values.

**Table 1.** Statistics of global representative average δ^13^C-CH_4_ values for the major CH_4_ categories used in the Monte Carlo simulations for 1993–2017. The interannual variability (IAV), uncertainty propagated from bottom-up estimates, and the full uncertainty that considers both uncertainty from emissions and spatially resolved distribution of source signatures for different CH_4_ categories are listed. See Table S2 for CH_4_ sectors that are not shown here.

δ^13^C-CH_4_ (‰)	Mean	IAV	Uncertainty from emissions	Full uncertainty
Coal	−45.8	0.71	1.23	2.57
Oil & gas	−43.8	0.06	0.61	0.93
Livestock	−65.4	0.16	0.32	0.86
Wetlands	−59.6	0.10	0.15	0.20
Biomass burning^a^	−23.9	0.38	0.03	0.14

^a^The higher IAV of BB_CH4_ than the uncertainty range is due to the spikes during some extreme El Niño years, which are one order of magnitude higher than that of most years.

**Figure 5.**
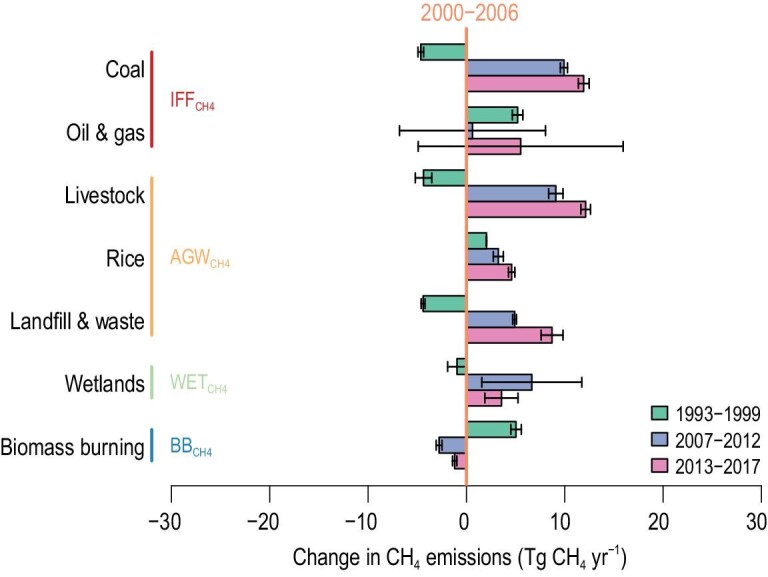
Changes in average total CH_4_ emissions in the most likely scenarios. The changes in CH_4_ emissions over the three periods are calculated relative to the average in the CH_4_ plateau period 2000–2006 (vertical reference line in orange). The most likely scenarios are defined as the subset of emission scenarios (*n* = 960) in the first percentile lowest mean squared difference (MSD) (Fig. S6) in comparison to the full ensembles (*n* = 96 000; Fig. S9). IFF_CH4_, AGW_CH4_, WET_CH4_ and BB_CH4_ represent the CH_4_ sectors of industrial fossil fuels, agriculture and waste, wetlands, and biomass burning, respectively.

We calculate the most likely scenarios based on the agreement of bottom-up estimates with isotopic observations (Fig. 5). The results suggest that the agricultural, landfill and waste sectors account for 53 ± 13% (21.0 ± 0.8 Tg CH_4_ yr^–1^; 1-σ) of renewed growth over the period of 2007–2017 compared to 2000–2006, with industrial fossil fuel sources and wetland sources contributing 34 ± 24% (13.7 ± 8.8 Tg CH_4_ yr^–1^) and 13 ± 9% (5.3 ± 3.5 Tg CH_4_ yr^–1^), respectively. The decreasing emissions from fossil fuel sectors in 1993–1999 compared to 2000–2006, combined with the increasing OH anomaly (Fig. S8), may have contributed to the CH_4_ stabilization period (Fig. 5 and Fig. S7). The increases in methane emissions (mainly from the fossil fuel, agriculture and waste sectors) combined with a step increase from wetland CH_4_ and small decreases in OH levels led to renewed growth in methane during 2007–2012. Moreover, the higher CH_4_ emissions from mainly anthropogenic activities, i.e. coal, oil, gas, livestock, landfill and waste sectors, drove the accelerated increase in atmospheric CH_4_ during 2013–2017. These sectoral emission increases are consistent with economic activity data (Table S2; Table S3) showing that, in the past decade, coal production has increased by 41.7% globally (International Energy Agency, https://www.iea.org/topics/coal/statistics/) and that the populations of major livestock species (e.g. swine, chickens and ruminant animals) have increased by 22.5% (FAOSTAT, http://www.fao.org/faostat/). Although coal production exhibited a temporary decline during 2014–2016 (Statistical Review of World Energy 2020, https://www.bp.com/content/dam/bp/business-sites/en/global/corporate/pdfs/energy-economics/statistical-review/bp-stats-review-2020-full-report.pdf), average coal emissions during 2013–2017 were higher than those during 2007–2012, indicating that coal mining emissions continued to grow, with a higher contribution to the increase in atmospheric CH_4_.

## CONCLUSIONS

Our analysis shows that a comprehensive evaluation of hypotheses regarding the attribution of rising atmospheric CH_4_ based on a combination of bottom-up approaches and isotopic values can reconcile multiple lines of evidence into a robust global CH_4_ budget. However, we acknowledge that there are some biases and uncertainties in the bottom-up estimates and that our exploration of possible emission scenarios does not cover all potential scenarios. This study clearly suggests that the proposed hypotheses are influenced by the choice of a priori estimates, indicating that the high-bias a priori estimates of trends applied in some earlier studies have led to equally biased conclusions regarding the attribution of atmospheric methane rise. Our results suggest that decreasing emissions from coal, oil and gas from 1993–1999 to 2000–2006, combined with the increasing OH anomaly, likely contributed to the methane stabilization period. Anthropogenic sources were the most likely major contributor to the renewed growth in CH_4_ after 2006. Moreover, the good agreement of low present-day geological source estimates with observations supports the hypothesis that the IFF_CH4_ in recent decades has been largely underestimated. However, our understanding of the role of livestock and wetlands, particularly in tropical regions, is more limited [58,59]. Aircraft measurements in these regions may help address the lack of data and improve our understanding of WET_CH4_. This study highlights the dominant role of anthropogenic emissions from fossil fuels, agriculture, landfills and waste in driving the recent rising trend in atmospheric CH_4_. Our findings improve our understanding of the causes of changes in atmospheric CH_4_ over the past 25 years, enabling the development of more targeted mitigation strategies and policies to stabilize and ultimately reduce key contributing emission sectors.

## MATERIALS AND METHODS

### Model descriptions

The model was developed from previous studies [15,60,61] and consists of two perfectly mixed boxes representing the troposphere in the northern and southern hemispheres. The changes in CH_4_ concentration are calculated using the following equations:

(1) \begin{eqnarray*} &&^{12}\!{\textit{ CH}}_4^N\!\!\left( {t + \Delta t} \right) = {}^{12}{\textit {CH}}_4^N\!\!\left( t \right) \nonumber\\ &&\quad + \left( \sum \limits_i {}^{12}S_i^N\!\!\left( t \right) + \sum \limits_j k_j^{12}{}^{12}{\textit {CH}}_4^N\!\!\left( t \right)\right.\nonumber\\ &&\quad\left. -\, \frac{1}{2{\tau _{ex}}}{}^{12}{\textit {CH}}_4^N\!\!\left( t \right) + \frac{1}{2{\tau _{ex}}}{}^{12}{\textit {CH}}_4^S\!\!\left( t \right) \vphantom{\left( \sum \limits_i {}^{12}S_i^N\!\!\left( t \right) + \sum \limits_j k_j^{12}{}^{12}{\textit {CH}}_4^N\!\!\left( t \right)\right.}\right){\Delta }t, \end{eqnarray*}

(2) \begin{eqnarray*} &&{}^{12}{\textit {CH}}_4^S\!\!\left( {t + \Delta t} \right) = {}^{12}{\textit {CH}}_4^S\!\! \left( t \right) \nonumber\\ &&\quad + \left( \mathop \sum \limits_i {}^{12}S_i^S\!\!\left( t \right) + \mathop \sum \limits_j k_j^{13}{}^{12}{\textit {CH}}_4^S\!\!\left( t \right) \right.\nonumber\\ &&\quad\left. - \frac{1}{{2{\tau _{ex}}}}{}^{12}{\textit {CH}}_4^S\!\!\left( t \right)+ \frac{1}{{2{\tau _{ex}}}}{}^{12}{\textit {CH}}_4^N\!\!\left( t \right)\vphantom{\left( \mathop \sum \limits_i {}^{12}S_i^S\!\!\left( t \right) + \mathop \sum \limits_j k_j^{13}{}^{12}{\textit {CH}}_4^S\!\!\left( t \right) \right.} \right){\rm{\Delta }}t,\nonumber\\ \end{eqnarray*}

where ^12^CH_4_ is approximated by CH_4_ and ^12^$S_i^N$(*t*) and ^12^$S_i^S$(*t*) represent the annual source strength of the source in the northern hemisphere and southern hemisphere, respectively. *k*^12^ is the first-order removal rate coefficient for the sinks. The interhemispheric exchange time τ is set to a constant value of 1 yr given that the overall methane CH_4_ concentration and OH anomalies are largely unaffected by the interhemispheric exchanges [15].

The δ^13^C-CH_4_ isotopic signatures of the different source categories *i* and the kinetic isotope effect (KIE) in the individual sink reactions *j* are used to calculate the sources (^13^*S*_i_) and removal rate coefficients (^13^*k*_j_) for δ^13^C-CH_4_ values.

These terms are then used to derive the mixing ratio changes in ^13^C-CH_4_:

(3) \begin{eqnarray*} &&{}^{13}{\textit {CH}}_4^N\! \left( {t + \Delta t} \right) = {}^{13}{\textit {CH}}_4^N\! \left( t \right) \nonumber\\ &&\quad + \left( \mathop \sum \limits_i {}^{13}S_i^N\!\!\left( t \right) + \mathop \sum \limits_j k_j^{13}{}^{13}{\textit {CH}}_4^N\!\!\left( t \right)\right. \nonumber\\ &&\quad\left. -\, \frac{1}{{2{\tau _{ex}}}}{}^{13}{\textit {CH}}_4^N\!\!\left( t \right) + \frac{1}{{2{\tau _{ex}}}}{}^{13}{\textit {CH}}_4^S\!\!\left( t \right)\vphantom{\left( \mathop \sum \limits_i {}^{13}S_i^N\!\!\left( t \right) + \mathop \sum \limits_j k_j^{13}{}^{13}{\textit {CH}}_4^N\!\!\left( t \right)\right.} \right){\rm{\Delta }}t,\nonumber\\ \end{eqnarray*}

(4) \begin{eqnarray*} &&{}_{\rm{\ }}^{13}{\textit {CH}}_4^S\!\!\left( {t + \Delta t} \right) = {}_{\rm{\ }}^{13}{\textit {CH}}_4^S\!\!\left( t \right) \nonumber\\ &&\quad + \left( \mathop \sum \limits_i {}_{\rm{\ }}^{13}S_i^S\!\!\left( t \right) + \mathop \sum \limits_j k_j^{13}{}_{\rm{\ }}^{13}{\textit {CH}}_4^S\!\!\left( t \right) \right.\nonumber\\ &&\left.\quad - \frac{1}{{2{\tau _{ex}}}}{}_{\rm{\ }}^{13}{\textit {CH}}_4^S\!\!\left( t \right) + \frac{1}{{2{\tau _{ex}}}}{}_{\rm{\ }}^{13}{\textit {CH}}_4^N\!\!\left( t \right)\vphantom{\left( \mathop \sum \limits_i {}_{\rm{\ }}^{13}S_i^S\!\!\left( t \right) + \mathop \sum \limits_j k_j^{13}{}_{\rm{\ }}^{13}{\textit {CH}}_4^S\!\!\left( t \right) \right.} \right){\rm{\Delta }}t.\nonumber\\\end{eqnarray*}

The mixing ratios of the individual isotopologues are converted to δ values as follows:

(5) \begin{equation*} \delta _C^{13} = \ \left( {\frac{{{}^{13}{\textit {CH}_4}/{}^{12}{\textit {CH}_4}}}{{{}^{13}{R_{\rm std}}}} - 1} \right), \end{equation*}

where ^13^*R*_std_ = 1.12372% is the ^13^C/^12^C ratio of the international reference material Vienna Pee Dee Belemnite (VPDB).

The soil sink is considered to have a low IAV, as suggested by biogeochemical models [62,63], despite a recent study [64] based on a few site-level measurements suggesting a decline in the soil sink in temperate forests in recent decades. For soil CH_4_ uptake, we use climatology from a process-based model [63] in the calculation of the hemispheric net CH_4_ source (see equation 6 in Materials and Methods). The contributions of Cl sink and stratospheric loss to the removal of CH_4_ in the troposphere are highly uncertain and not well constrained by direct observations, but have a strong kinetic isotope effect on ^13^CH_4_. Given the large uncertainty in Cl and stratospheric sinks and the lack of available datasets, the magnitudes of these two sinks were not explicitly considered in the calculation of the hemispheric CH_4_ budget. We assume that the annual methane removal rate is driven solely by OH variability, while other minor sinks are kept constant over the study period. Because sensitivity tests [65] suggest that the uncertain magnitude of Cl fields leads to a wide range of simulated δ^13^C-CH_4_ values given its strong ‘isotope leverage’ effect [66] (−60 ± 1‰) on total ϵ, the sink-weighted average fractionation factor ϵ is highly uncertain. The approach in this study is to estimate the total ɛ value for each box model run that optimizes the match between atmospheric observations and simulation at the onset of the study period. The optimized ɛ values were derived from running the box model in inverse mode by matching the observed global average [11]. This allows us to explore the uncertainty in ɛ based on bottom-up source aggregation and the uncertainty in δ^13^C-CH_4_ values. Figure S6 shows that the estimated fractionation factors for the full ensemble and first percentile ensemble are broadly in agreement with previous studies [11–13,60,66–70]. The distribution of the mean methane lifetime (Fig. S9) over the study period is slightly lower than the estimated 9.1 ± 0.9 yr from Ref. [71] and is comparable in magnitude to that between atmospheric chemistry-transport models in the recent model intercomparison [31,32,72]. Here, we evaluate the global results from the box model, instead of hemispheric results, to minimize the potential influence of uncertainty in IAV from interhemispheric transport on box model performance, as suggested by a recent study [73]. See Supplementary Data for details about the model strategy.

### CH_4_ source estimates

To test all the proposed competing hypotheses, we carried out simulation experiments using box modeling for different emission scenarios based on a suite of bottom-up datasets. We first list all the possible options for the CH_4_ inventories by five CH_4_ source categories (i.e. IFF_CH4_, AGW_CH4_, WET_CH4_, BB_CH4_ and GEO_CH4_) and then generate emission scenarios with combinations of CH_4_ inventories. The assignment of the inventory (i.e. EDGAR)-specific sectors into the main categories IFF_CH4_, AGW_CH4_ and BB_CH4_ follows the criteria from Supplementary Table S4 in Ref. [5]. Anthropogenic CH_4_ emissions related to fossil fuels from exploitation, transportation and usage of coal, oil and natural gas are defined as IFF_CH4_. For methane sectors related to enteric fermentation and manure, landfills, waste and rice agriculture are defined as AGW_CH4_.

### Spatially resolved **δ**^13^C-CH_4_ and uncertainty estimation

Spatially resolved distributions of δ^13^C-CH_4_ source signatures for the following major methane categories were applied in this study: coal, natural gas/oil, livestock, wetlands and biomass burning. For the other sources, including agricultural waste, rice, geological sources, termites, freshwater systems and wild animals, we use a globally averaged value (Table S4) from a global inventory database that collected isotopic source signatures based on literature values [36,66,68].

### Emission scenarios

An emission scenario is a combination of the individual CH_4_ source estimates listed in Table S1. Annual total net CH_4_ sources can be expressed as follows:

(6) \begin{eqnarray*} {S_{{\rm tot}}} &=& {S_{{\rm IFF}}}\ + {S_{{\rm AGW}}} + {S_{{\rm WET}}} + {S_{{\rm BB}}} + {S_{{\rm GEO}}},\nonumber\\ && +\, {S_{{\rm OTH}}} - {S_{{\rm soil}}}, \end{eqnarray*}

where *S* represents the individual CH_4_ source from Table S1 and *S*_soil_ is a constant soil sink. The total number of emission scenarios is 96, calculated as 4 IFF_CH4_ × 3 AWG_CH4_ × 2 WET_CH4_ × 2 BB_CH4_ × 2 GEO_CH4_. For each emission scenario, we use Monte Carlo techniques to estimate the uncertainty in the source signature propagated from bottom-up estimates and the spatial variability of the source signature. A set of 1000 random maps of δ^13^C-CH_4_ values for each major CH_4_ source (Table 1) were generated based on the uncertainty maps in this study assuming a Gaussian distribution. For CH_4_ sources that are not spatially resolved, 1000 samples of the global-representative signature values are calculated with mean and 1-standard deviation defined by observations from the compiled databases (Table S4). One thousand sets of emission-weighted hemispheric time series of δ^13^C-CH_4_, which were calculated with bottom-up estimates depending on emission scenarios, were used as inputs for the box model. For each emission scenario, the simulated time series of δ^13^C-CH_4_ values covers the uncertainty range of spatial variability in the isotopic signatures of major CH_4_ categories.

## DATA AVAILABILITY

All data needed to evaluate the conclusions in this paper are present in the paper and/or the Supplementary Data. Additional ancillary data are available from the corresponding author upon request.

## Supplementary Material

nwab200_Supplemental_File
