# Supplementary material for: Anthropogenic emission is the main contributor to the rise of atmospheric methane during 1993–2017
Source: Natl Sci Rev. 2021 Nov 11;9(5):nwab200. doi: 10.1093/nsr/nwab200 (PMC9084358; doi:10.1093/nsr/nwab200)
Supplement: nwab200_Supplemental_File [file nwab200_supplemental_file.docx]

**Supplementary Information for**

Anthropogenic emissions are the main contribution to the rise of atmospheric methane (1993-2017)

Zhen Zhang (张臻)^1*^, Benjamin Poulter^2^, Sara Knox^3^, Ann Stavert^4^, Gavin McNicol^5^, Etienne Fluet-Chouinard^6^, Aryeh Feinberg^7^, Yuanhong Zhao (赵园红)^8^, Philippe Bousquet^9^, Josep G. Canadell^4^, Anita Ganesan^10^, Gustaf Hugelius^11^, George Hurtt^1^, Robert B. Jackson^6,12^, Prabir K. Patra^13^, Marielle Saunois^9^, Lena Höglund-Isaksson^14^, Chunlin Huang (黄春林)^15^, Abhishek Chatterjee^16,17^, Xin Li (李新)^18^

*Corresponding author: Zhen Zhang. **Email:** [yuisheng@gmail.com](mailto:yuisheng@gmail.com)

# List of Acronyms

EDGAR                 Emission Database for Global Atmospheric Research

WOLF2017 Agricultural CH_4_ sources includes Inventories for Livestock CH_4_ from Wolf et al., (2017) and Rice and Waste sectors from EDGARv4.3.2

GAINS GHG-Air pollution INteraction and Synergies model (GAINS)

SCH150 Industrial Fossil Fuel inventories from Schwietzke et al., (2016)

LPJ                       Lund-Potsdam-Jena Dynamic Global Vegetation Model

CRU              Wetland CH_4_ estimate using meteorological forcing from Climate Research Unit, representing small increases in wetland CH_4_ due to changing climate

REN                            Wetland CH_4_ estimate using an ensemble of meteorological forcing from Reanalysis, representing step increases in wetland CH_4_ due to enhanced precipitation and rising temperature

BB4CMIP            Historic global biomass burning emissions for CMIP6

GFED Global Fire Emissions Database

IAV                       Interannual Variability

MCF                 Methyl chloroform

IFF_CH4_                   Industrial Fossil Fuel CH_4_ emissions

COA_CH4_ Coal CH_4_ emissions

GAS_CH4_ Natural Gas/Oil CH_4_ emissions

AGW_CH4_                    Agriculture and Waste CH_4_ emissions

RIC_CH4_ Agricultural Rice CH_4_ emissions

WAS_CH4_ Waste/Landfill CH_4_ emissions

LIV_CH4_ Livestock CH_4_ emissions

BB_CH4_                    Biomass Burning CH_4_ emissions

WET_CH4_                    Wetland CH_4_ emissions

GEO_CH4_ Geological CH_4_ emissions

OH                        Hydroxyl Radical

δ^13^C-CH_4_                    ^13^C/^12^C-ratio in CH_4_

ɛ Isotopic fractionation factor

**1. Modeling strategy**

The model was first run for 1980-2017 in inverse mode with CH_4_ emissions, emission-weighted average δ^13^C-CH_4_ values for the sources, and atmospheric CH_4_ and δ^13^C-CH_4_ observations [1,2] to derive historic time series of global OH for northern and southern hemisphere and a constant fractionation factor ε for each run of the specific emission scenarios. In this inverse mode, the fractionation factor ε was adjusted to match the source δ^13^C-CH_4_ values for each run, with the optimized δ^13^C-CH_4_ value at the onset of the study period (i.e., 1992) using a bisection method, yielding the so-called baseline runs. The derived time series of OH and fractionation factor ε were then used for the perturbation runs in the forward mode. Note that the OH time series were calculated by balancing the sources with observations, which were not optimized by observational constraints (e.g., MCF) nor constrained by any mechanical processes.

For the baseline runs, the box model can, by design, reproduce the atmospheric history in both CH_4_ concentration and δ^13^C-CH_4_ value for each run of the emission scenarios. For the perturbation runs, to examine the study period, the source δ^13^C-CH_4_ values calculated from Monte Carlo calculations of corresponding emission scenarios were used as inputs. The resulting modeled δ^13^C-CH_4_ value was evaluated against the observed δ^13^C-CH_4_ value to quantify the biases in the emission scenarios. The assumption for our modeling design is that, due to the large uncertainty and limited understanding of the IAV in OH, the year-to-year changes in atmospheric CH_4_ for most of the prior assumptions of the total CH_4_ source can be explained by any prescribed variations in the methane removal rate.

The bias in the simulated δ^13^C-CH_4_ value may come from a single source type or a combination of concurring or compensating sources. The approach of generating a large ensemble of emission scenarios based on different bottom-up estimates implicitly covers the uncertainty in the CH_4_ source signature and emission magnitudes. This provides explanations based on mechanisms for attributing the changes in CH_4_ sources. The box model was first initialized in 1700 and for the period of 1700-1980 with prescribed anthropogenic emissions and climatology of natural emissions [3] as the first spin-up phase. The simulations were then run with emission scenario-specific sources for the 1980-1992 period for a second spin-up. The study period is from 1993-2017. These choices avoid the artificial shift in δ^13^C-CH_4_ value due to spin-up and remove disruptions by the Mt. Pinatubo eruption.

Table S1 provides a summary of the data sources used in generating CH_4_ source emission scenarios. The time series of hemispheric δ^13^C-CH_4_ values is calculated as the emission-weighted average δ^13^C-CH_4_ of all CH_4_ sectors with a consideration of the spatial variability in δ^13^C-CH_4_ values for major CH_4_ sectors. The spatial distributions of δ^13^C-CH_4_ values were generated at the subcategory level (Table S1). The annual total hemispheric emissions were calculated as the sum of each CH_4_ source category. For simplification, we assume constant CH_4_ emissions from the other natural sources, including freshwater systems, wild animals, termites, geological sources, and other minor natural sources over the study period.

**2. Processing of CH_4_ sources**

**Industrial fossil fuels.** We use global IFF_CH4_ estimates from four datasets, EDGARv4.2, EDGARv4.3.2, GAINS Eclipse v6, and SCH150. EDGAR is a bottom-up inventory dataset based on the energy balance statistics of International Energy Agency reports, while SCH150 was a gridded anthropogenic CH_4_ emission dataset derived from an atmospheric inversion model simulation [4] in which IFF_CH4_ was approximately assumed to be in the upward-revised percentile range (~ 150 Tg CH_4_ yr^-1^ in total) using coal, natural gas/oil, and other industrial sources [5] in an atmospheric transport model TM5 simulation. The IFF_CH4_ in this study comprises the CH_4_ emissions from the energy, fugitive, and industrial process categories, which do not distinguish between shale gas and conventional natural gas. Here, EDGARv.4.2 represents a harmonized gridded time series merging the original EDGARv4.2, an extended gridded product EDGARv4.2FT2010, and an extended time series EDGARv.4.2FT2012. We assume that the yearly gridded emissions have a small IAV in spatial variability during 2010-2012 and that the increase in IFF_CH4_ is proportional to the increases in total anthropogenic CH_4_ emissions. The CH_4_ emissions after 2010 are set up using the spatial distribution of the mean EDGARv4.2 over 2008-2010. The yearly gridded maps for 2011 and 2012 were calculated by scaling the mean of 2008-2010 to match EDGARv4.2FT2012 (gridded map times the ratio between total emissions in EDGARv4.2FT2012 and total emissions in EDGARv4.2). EDGARv4.3.2 suggests that IFF_CH4_ declined in the 1970s and then stabilized in the 1980s and 1990s, while EDGARv4.2 shows a constant increase during the 1990s and 2000s. We treated EDGARv4.2 and EDGARv4.3.2 as separate datasets, as the temporal pattern of IFF_CH4_ in the new version EDGARv4.3.2 was largely revised due to updated emission factors and statistical methodology, thus reflecting different hypotheses for explaining the changes in IFF_CH4_ during the 1980s and 1990s. The GAINS model [6] of the International Institute for Applied Systems Analysis (IIASA) is a multipollutant emission estimation model with bottom-up emissions estimates and future mitigation potentials for any externally given energy sector scenario. For this particular exercise, the global energy balance statistics of the International Energy Agency were used. It makes use of country-specific information on associated petroleum gas generation, recovery and venting/flaring rates, with the latter calibrated to satellite image estimates of volumes of gas flared.

**Agriculture and waste.** EDGARv4.2, EDGARv4.3.2, and WOLF2017 all use a bottom-up methodology that summarizes the livestock population, country-specific statistics, and emission factors. WOLF2017 represents the sum of livestock CH_4_ emissions from [7] and rice and landfill/waste emissions from EDGARv4.3.2. WOLF2017 estimates systematically higher livestock emissions based on updated emission factors due to reported recent changes in animal body mass, feed quality and quantity, and management of livestock and manure. Livestock emissions are the largest source in the AGW_CH4_ categories, contributing an average of 57% to the total across the three livestock inventories over the study period, while the rice and landfill/waste sectors contribute 21% and 22%, respectively. In addition, the total AGW_CH4_ increased from 181 Tg CH_4_ yr^-1^ to 202 Tg CH_4_ yr^-1^ from 2000-2006 to 2007-2017, with livestock, rice agriculture, and landfills/waste contributing 50%, 19%, and 31% of the total increase, respectively (Fig. 1C). Changes in livestock populations play a large role in the magnitude of CH_4_ fluxes associated with livestock. Given the similar trend in AGW_CH4_ in both versions of EDGAR estimates, the trend from EDGARv4.3.2 was scaled to match WOLF2017 in 2000 to derive the time series of the study period for WOLF2017.

**Wetlands**. We use the land surface model LPJ-wsl to simulate the spatiotemporal patterns of wetland CH_4_ emissions. LPJ-wsl is a process-based dynamic global vegetation model developed for carbon cycle applications, which includes water/carbon processes, vegetation demography and dynamics that are represented by plant functional types (PFTs). The CH_4_ emissions are modeled as a function of wetland area, heterotrophic respiration (Rh), and scaling factors that represent soil temperature and moisture effects on methane production in different biomes.

The wetland area and dynamics were simulated using a topography-based hydrological model (TOPMODEL) with a prescribed high-resolution topographic index product, hydrological data and maps based on global SHuttle Elevation Derivatives at multiple Scales (HydroSHEDS) [8]. The CH_4_ emissions simulated by LPJ-wsl have been validated against observations at the regional and global scales[9,10].

To take into account the uncertainty in estimated wetland CH_4_ emissions from climate variables, we applied four wetland CH_4_ estimates based on LPJ simulations forced by four climate datasets: one observational geostatistical dataset from the Climate Research Unit (CRU) and three state-of-the-art meteorological reanalysis datasets, including 1-hourly reanalysis Modern-Era Retrospective analysis for Research and Applications Version 2 (MERRA2) from the NASA Global Modeling and Assimilation Office, 6-hourly ERA-Interim from the European Centre for Medium-Range Weather Forecasts data assimilation system, and 6-hourly Japanese 55-year Reanalysis (JRA-55) from the Japan Meteorological Agency. The ensemble mean of CH_4_ emissions from simulations with reanalyses was applied in this study (denoted REN). Both of the wetland CH_4_ estimates cover the 1980-2017 period [11].

In general, LPJ-wsl suggests a step increase in CH_4_ emissions by 7.3±1.6 Tg CH_4_ yr^-1^ during 2007-2017 relative to the period of 2000-2006 when using the meteorological reanalysis, while the CRU-based simulation found a minor increase in CH_4_ emissions between these two periods. This step increase is mainly caused by the expanding wetland areas in the tropics due to enhanced precipitation, with a positive trend of 0.8 Tg CH_4_ yr^-1^ yr^-1^ for 2000-2017. This positive trend found in the tropics is generally larger than the WetCHARTs estimates but lower than the recent atmospheric inversions [12–14] of GOSAT data, which suggest a positive trend of 1.5-2.1 Tg CH_4_ yr^-1^ yr^-1^ in the region for 2010-2018.

**Biofuel and biomass burning.** Two datasets were applied to represent hypotheses for BB_CH4_: GFED (version 4.1s) [15] and a satellite-based estimate [16] (denoted Worden2017). GFED represents the conventional view that has been widely applied in atmospheric models. Worden2017 is one recent estimate based on inversion modeling of the Terra Measurement of Pollution in the Troposphere (MOPITT) satellite observations and inventories. Worden2017 is a revised BB_CH4_ dataset that uses the same burned area from GFED v4.1 but uses CO flux estimates based on MOPITT retrievals and the atmospheric transport model GEOS-CHEM to constrain the CH_4_ emission factors, which showed a higher decrease in BB_CH4_ than BB4CMIP during 2000-2012. Both datasets are subject to uncertainty in the estimates of the burned area as a proxy for biomass burning. We use BB4CMIP, which applies the GFED mapped burned area with a fractional contribution of different fire types from terrestrial ecosystem fires, agricultural waste burning (e.g. crop waste fires), and deforestation and degradation with estimates starting from 1750, to complete the full-time series for years before 1997 for Worden2017. Biofuel emissions from EDGARv4.2 are applied to generate GFED-based and Worden2017-based BB_CH4_ scenarios to make them consistent with the setup in [16] (see Table S1).

**Geological sources.** Geological sources include onshore and offshore CH_4_ emissions. We tested two hypotheses for geological CH_4_ emissions based on recent debates [17,18] regarding geological methane: one scenario hypothesizes that natural geologic sources account for a substantial component of the atmospheric methane budget [19], and the other hypothesizes that contemporary natural GEO_CH4_ emissions are overestimated according to paleoradiocarbon evidence [17]. In the former scenario, GEO_CH4_ is estimated to have a mean value of 52 Tg CH_4_ yr^-1^, while in the latter scenario, GEO_CH4_ is assumed to be 15 Tg CH_4_ yr^-1^, which is a conservative value with 95% confidence of the estimate [17]. Note that this hypothesis suggests that the contemporary geologic source is likely less than 15 Tg CH_4_ yr^-1^ due to more erosion associated with the lower sea level in the late glacial period. The deducted GEO_CH4_ in the latter hypothesis was added to the gas/oil sector of IFF_CH4_ by scaling IFF_CH4_ with a factor to balance the methane budget. This treatment was made based on evidence from several recent studies, which suggest that the current natural fossil fuel CH_4_ emissions (i.e., geological seepages) are overestimated, and industrial fossil fuels are underestimated [4,17,20]. The GEO_CH4_ in both scenarios is set to be constant with values of 52 Tg CH_4_ yr^-1^ and 15 Tg CH_4_ yr^-1^ for the former and latter attribution scenarios, respectively, in this study, assuming that GEO_CH4_ has no IAV during the time period, as suggested by previous studies [1,21]. The hemispheric total of GEO_CH4_ is calculated based on a gridded map of geological methane emissions [22].

**Other sources.** The other sources include freshwater ecosystems, termites, oceanic hydrate sources, wild animals, permafrost, and potential vegetation sources. In recent syntheses [23,24], the estimate for freshwater systems has been revised to a range of 99-272 Tg CH_4_ yr^-1^ (mean of 185 Tg CH_4_ yr^-1^). However, this is likely an overestimate, as there is generally double-counting in bottom-up estimates of freshwater systems (e.g. lakes, rivers, and ponds) and global wetlands and an overestimation in other sources, such as vegetation. Double counting occurs because, for freshwater ecosystems, the prior estimates used for parameter calibration are usually derived from the inversion model, which does not distinguish freshwater systems and wetlands. The use of inversion models is necessitated in part by the large uncertainties associated with regional upscaling of site-level observations of highly variable CH_4_ fluxes. In addition, the inland freshwater distribution (e.g. lake, ponds, and rivers) applied in the upscaling [25,26] are potentially overlapped with the distribution in the wetland CH_4_ estimates due to the difficulty in accurate splitting between wetland and freshwater extent [27]. Here, we assume that the total CH_4_ emissions from freshwater systems, wild animals, and other residual natural sources are constant at 61 Tg CH_4_ yr^-1^ (with a value of 9 Tg CH_4_ yr^-1^ from termites, 40 Tg CH_4_ yr^-1^ from freshwater, 10 Tg CH_4_ yr^-1^ from wild animals and 2 Tg CH_4_ yr^-1^ from permafrost) for the purpose of balancing the global CH_4_ budget and simplification.

**Extrapolations of bottom-up estimates.** As this study aimed to investigate the variation in atmospheric CH_4_ from 1993-2017, we built extrapolated estimates for EDGARv4.2, WOLF2017, SCH150, and Worden2017 applied in this study up to 2017 using two statistical datasets: i) FAOSTAT emissions for the CH_4_ sectors of enteric fermentation, manure management and rice cultivation and ii) BP statistical review of fossil fuel production and consumption (<http://www.bp.com>, last access Aug. 28 2019) for the CH_4_ sectors of coal, oil, and gas. The extrapolation follows the data-driven approach applied in [28], and we use the same extrapolated datasets EDGARv4.3.2 and GAINS inventories from a recent synthesis [24]. For CH_4_ sectors in EDGARv4.2 (i.e., COAL, OIL, GAS, RIC, MNM (Manure), and ENF (Enteric)), WOLF2017 (MNM and ENF), SCH150 (COAL, OIL, and GAS), where FAO and BP statistics have estimates, the methane emissions for year *t* are set up to equal the emissions in 2012 times the ratio between the emissions of the reference dataset (i.e., 0.5 FAOSTAT/BP) in year *t* (*E_REF_*) and the emissions of the reference dataset in 2012. For each emission sector *i*, the region-specific emissions using a region map defined in Regional Carbon Cycle Assessment and Processes (RECCAP) for the extrapolated inventories (E_EXT_) in year *t* are estimated as follows:

$E_{EXT}^{i}\left( t \right)=E_{EXT}^{i}\left( 2012 \right)\times(\frac{E_{REF}^{i}\left( t \right)}{E_{REF}^{i}(2012)})$ (1)

For CH_4_ sectors (i.e., ENE (energy), FFF (fossil fuel fires), TNR (transport), TRO (road transport), IND (industry), SWD (solid waste), WWT (wastewater), RCOP (biofuel), and AWB (agricultural waste burning)) in EDGARv4.2 where the FAO and BP datasets do not exist, we calculate the ratio between the regional totals of the reference year (i.e., 2012) and the last 3 years of data (2010-2012) and then linearly propagate the ratios for 2013-2017. For the biomass burning sector, we use the burned area from GFED4.1 and the CH_4_/CO ratio from the last three years of data (2010-2012) in the Worden2017 dataset to extend Worden2017 up to 2017 assuming a linear trend in the CH_4_/CO ratio.

**3. Processing of δ^13^C-CH_4_ in source**

**Industrial fossil fuel δ^13^C-CH_4._** The calculation of the coal δ^13^C-CH_4_ value was made using country-level coal values from a database [29], which provides coal isotopic values from 13 countries based on 1402 empirical samples, by averaging available measurements from coal mines in those countries. For the regions that do not have measurements available, we applied the mean coal values calculated from [4]. We use the country-level natural gas/oil δ^13^C-CH_4_ values from [29] and [4] to include the consideration of spatial variability in δ^13^C-CH_4_ values. The isotopic value map is based on measurements from 43 countries, accounting for ~ 70% of natural gas/oil emissions from 2000-2010. We applied the global mean calculated by [4] wherever data were unavailable.

**Livestock δ^13^C-CH_4._** We use the C3/C4 fraction map [30] to calculate regional livestock values. We assume that livestock animals graze or are fed vegetation that corresponds to the local resources and thus ignore the impact of the feedlots and imported fodder on the calculation of livestock δ^13^C-CH_4_ values. From the studies compiled by [29], the mean isotopic values of CH_4_ emitted by C3- and C4-fed livestock are -68.4‰ and -54.5‰, respectively. By weighting these two endmember signatures by the C3 and C4 fractions, we determined the spatial distribution of livestock δ^13^C-CH_4_ values [31].

**Wetland δ^13^C-CH_4._** The source signature map for wetlands was derived from [32], which was generated based on the literature for different wetland subtypes (e.g., bog, fen, and mineral wetlands with C3/C4 pathways). The signature map was evaluated against wetland signatures derived from atmospheric observations. See [32] for more details.

**Biomass burning δ^13^C-CH_4._** We assigned C3 and C4 biomass burning source values of -26.7‰ and -12.5‰ according to [29], based on the measurement of 965 plant material samples. We used the spatial distribution of the C3/C4 vegetation fraction to determine the locally mixed value and generated an isotopic map of biomass burning. Note that a recent study [33] suggests that samples of C4 are critically lacking and that the C4 signature should be revised to -17.9‰ given recent new measurements. We compared these two values with the emission estimate (Fig. S10), which suggests that applying the revised C4 value would yield a systematic shift in the BB_CH4_ source signature by ~0.4‰ in the negative direction. The results show that it does not affect the interannual variations in the emission-weighted signature of BB_CH4_ and thus has no impact on our conclusions.

**Other sources.** The other sources using a constant value in the isotopic signature calculation include landfills/waste, rice agriculture, freshwater systems, termites, wild animals, and permafrost and oceanic sources. These sources were treated in this way because there were either insufficient measurements to represent spatial distributions (e.g., freshwater systems and wild animals) or they did not have strong spatial variability due to the low heterogeneity in the CH_4_-generating environments (e.g., rice and termites). Given the similar underlying methanogenic processes and potential overlap in spatial distribution between freshwater systems and wetlands, the unweighted δ^13^C-CH_4_ values for freshwater systems were set to -61.5‰ as the global mean of wetland δ^13^C-CH_4_ values from [29]. The δ^13^C-CH_4_ values of other sources are listed in Table S2.

**Uncertainty maps.** The spatial distribution of uncertainty in wetland δ^13^C-CH_4_ values was generated based on four wetland types: boreal bog, fen, and mineral C3/C4 wetlands, where each type has an average signature and 1-σ uncertainty derived from [29]. The latitudinal gradients of wetland δ^13^C-CH_4_ values (Fig. S5) are broadly consistent with those of previous studies [34,35]. Each pixel is sampled with a given standard deviation assuming a Gaussian distribution. To calculate the C3/C4 mineral wetland signature, a climatology of wetland fraction map [36] and a C3 and C4 vegetation distribution map [30] were used to determine the distribution of wetland types and to calculate the average value.

For the uncertainty in the coal isotopic signature map, we used country-based estimates for the standard deviation given by [29]. The standard deviations in each country’s isotopic signature were calculated by [29], representing the spread of compiled in situ measurements. For all areas outside of the 13 countries where data are available, we apply the standard deviation of the global mean coal value (0.9‰). Similarly, for the natural gas isotopic signature map, we use the standard deviations for the 43 countries where measurements are available. Elsewhere, the standard deviation is set to the global natural gas isotopic signature standard deviation (0.6‰).

The livestock and biomass burning signature maps were both calculated using the weighted average of two endmember signatures and the C3 and C4 fractions from [30]. We neglected the uncertainty in the C4 fraction map, which excludes the effect of imported fodder on the C4 fraction map due to a lack of available datasets. We obtain isotopic signature uncertainty maps by propagating the standard deviation in the C3 and C4 isotopic endmembers, which is given by [29]. The following equation can be used to calculate the isotopic standard deviation (*σ^i^_13C_*) in a certain grid box *i*, with f*^i^_C4_* representing the C4 fraction in a certain grid box, f*^i^_C3_* representing the C3 fraction, and *σ_C4_* and *σ_C3_* representing the isotopic values of the endmembers.

$\sigma_{13C}^{i}=\sqrt{{(f_{C4}^{i}\times\sigma_{C4})}^{2}+{(f_{C3}^{i}\times\sigma_{C3})}^{2}}$ (2)

**4. Evaluation metrics**

Taylor diagrams [37] were used to visually evaluate the relative skill based on model-data fit by comparing the linear correlation, coefficient, RMSE, and the standard deviation in a polar coordinate plot. Additionally, to provide a single metric that evaluates the relative skill in reproducing the observations, the MSD was used to quantitatively rank the model performance. MSD is an integrated metric that describes the linear sum of the squared bias (SB), squared difference between model and observed standard deviations (SDSD), and the lack of correlation weighted by standard deviation (LCS). The MSD is the linear combination as follow:

$MSD=SB+SDSD+LCS$ (3)

The SB is the difference between model (x) and observation mean (y):

$SB={(\bar{x}- \bar{y})}^{2}$ (4)

The SDSD is the difference between the model (SD_m_) and observation (SD_s_) standard deviations, and a large value indicates that the model fails to simulate the amplitude of IAV:

$SDSD={({SD}_{s}- {SD}_{m})}^{2}$ (5)

The LCS term reflects the lack of correlation (r) between observations and models and is weighted by standard deviations:

$LCS=2{SD}_{s}{SD}_{m}(1-r)$ (6)

We carried out a sensitivity test for the selection of the subset of the emission scenarios that have the lowest bias relative to the observations by the thresholds of the 1^st^, 5^th^, and 10^th^ MSD percentiles. The probability density distributions of MSD at different thresholds are presented with the counting of box model runs for specific scenarios in Fig. S3, Fig. S11 and Fig. S12. The subset of emission scenarios within the 1st percentile has better agreement with the observed declines in δ^13^C-CH_4_ values for the post-2007 period and less bias with the OH time series from [38] than the full ensembles (Fig. S7). The results suggest that selecting the 1^st^ percentile can generate a significant improvement in simulating δ^13^C-CH_4_ values and in discriminating the scenarios for all emission categories for coal, natural gas and oil, livestock, and biomass burning, whereas EDGARv4.2 is excluded in the 1st and 5th percentile emission scenarios. The uncertainty ranges in Fig. 5 were calculated based on the uncertainty in individual sources and the uncertainty in the proportion of individual sources to the total source using error propagation equations.

**Datasets Used:** All the datasets used are publicly available. CH_4_ observations are from National Oceanic and Atmospheric Administration’s Earth System Research Laboratory (NOAA/ESRL) at ftp://aftp.cmdl.noaa.gov/data/trace_gases/ch4/flask/. ẟ^13^C-CH_4_ observations are from NOAA/ESRL at ftp://aftp.cmdl.noaa.gov/data/trace_gases/ch4c13/flask/, University of Washington, Seattle; University of Heidelberg at http://www.iup.uni-heidelberg.de/institut/forschung/groups/kk/Data_html; and University of California, Irvine, CA. The anthropogenic CH_4_ emissions EDGAR (v4.2 and v4.3.2) used in this study are available at https://edgar.jrc.ec.europa.eu/. The CH_4_ inventory datasets GAINS and SCH150 are included in the published articles by Höglund-Isaksson et al. (2017), Höglund-Isaksson et al. (2020) and Schwietzke et al. (2016). The revised livestock CH_4_ dataset from Wolf et al. (2017) is available at https://daac.ornl.gov/cgi-bin/dsviewer.pl?ds_id=1329. The GFED datasets are publicly available from the GFED website at https://www.globalfiredata.org/. The BB4CMIP dataset is available at http://www.globalfiredata.org/ar6historic.html. The ẟ^13^C-CH_4_ source signature databases are available at https://www.esrl.noaa.gov/gmd/ccgg/arc/?id=123 and https://www.esrl.noaa.gov/gmd/ccgg/arc/index.php?id=130. The coal production data are available from International Energy Agency at https://www.iea.org/topics/coal/statistics/. The FAOSTAT data on the populations of major livestock species are available from the FAO website at http://www.fao.org/faostat/en/#data. Additional ancillary data are available from the corresponding author upon request.


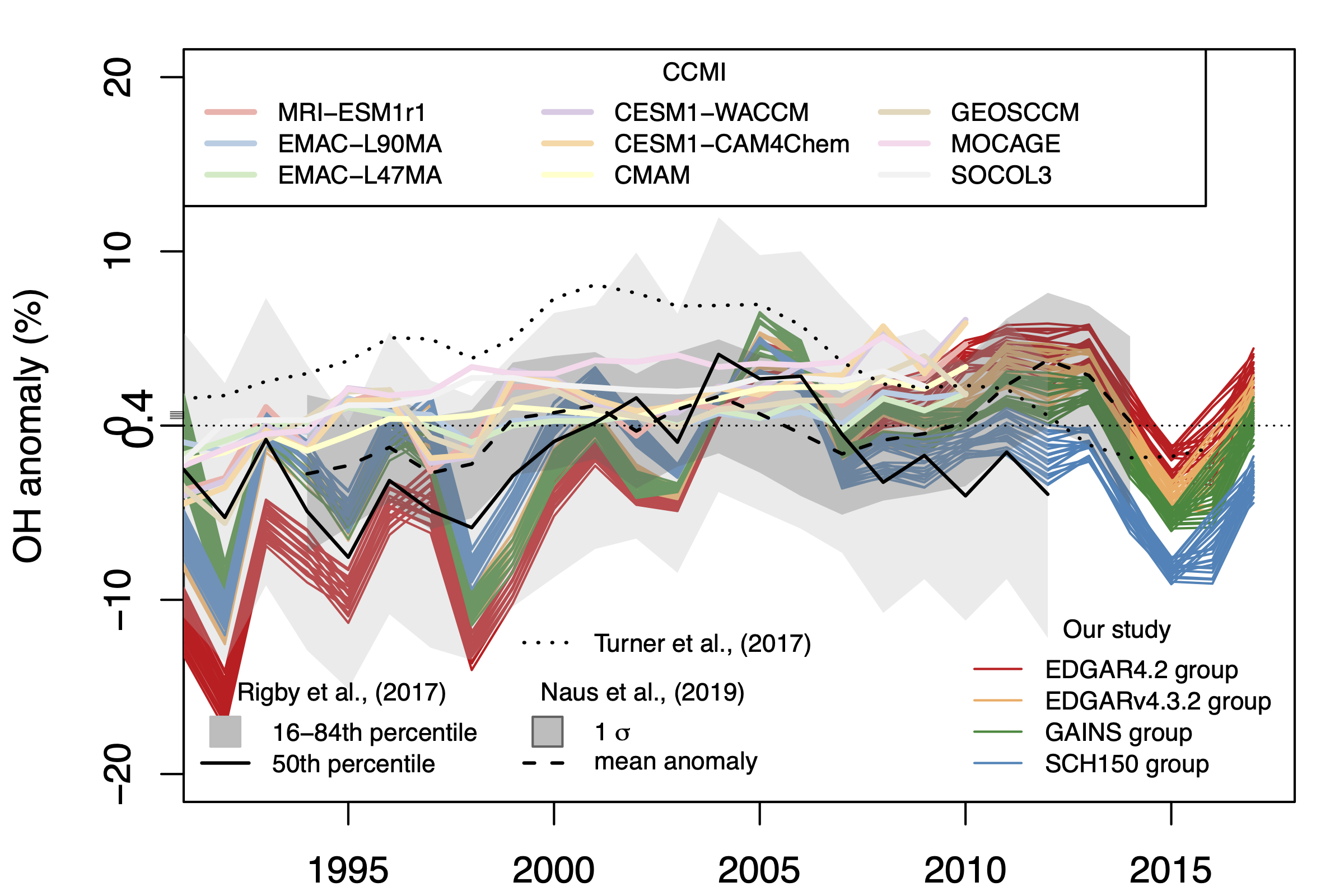


**Fig. S1. Inferred anomaly of tropospheric annual mean OH concentration by running the box model in inverse mode.** The black line shows the medians, and the shaded area shows ±1 sigma from methylchloroform (MCF)-based box model estimates[34]. The OH anomaly is calculated relative to a global mean concentration of 1e^6^ molecules (molec)/cm^3^. The dashed line represents an optimized box model estimate with bias corrections on constraints on OH[35]. In contrast to our study and the MCF-based estimates using the box model, the OH computed by a set of atmospheric chemistry models from the Chemistry Climate Model Initiative (CCMI) experiment[36] shows low interannual variability and low year-to-year fluctuation on the order of 1%.


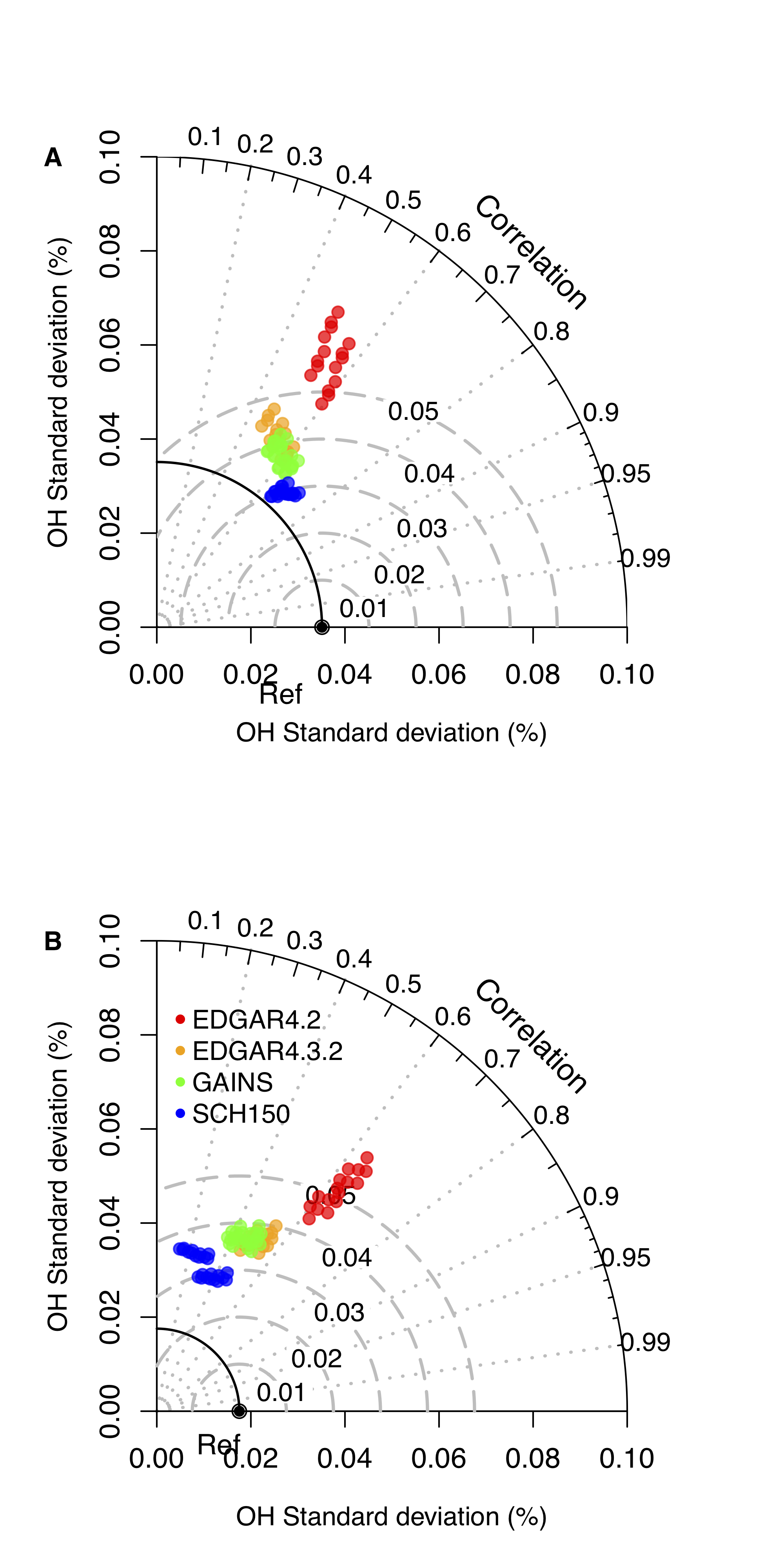


**Fig. S2. Performance of emission scenarios in simulating OH.** Taylor diagram illustrating the normalized standard deviation and Pearson’s correlation coefficient for the comparison of the OH anomaly (%) of different emission scenarios grouped by IFF_CH4_ with (A) the medians of OH anomalies inferred from the MCF-based estimates[36] and (B) means from an optimized box model estimate [37] (denoted ‘Ref’).


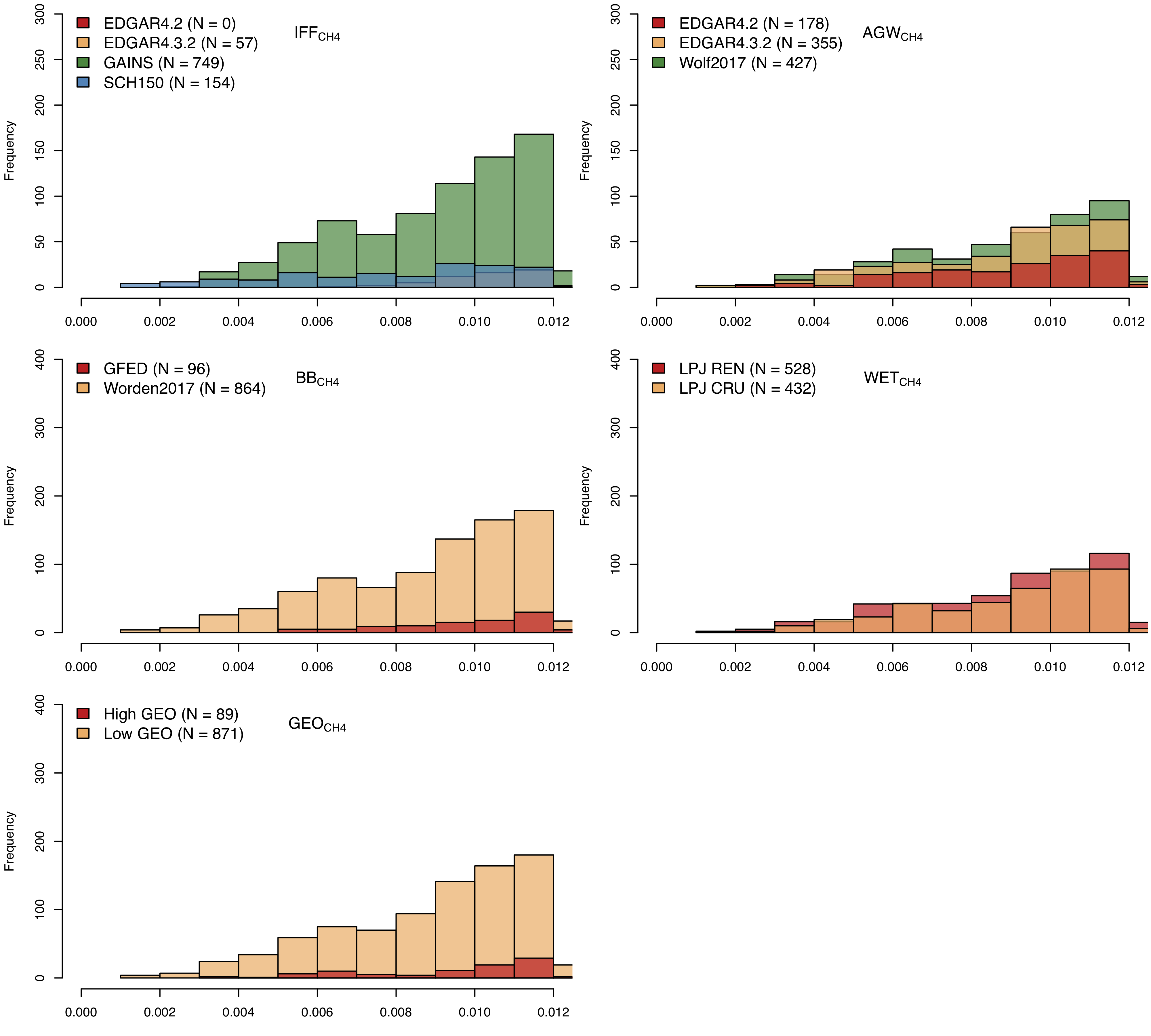


**Fig. S3. The histogram of the 1^st^** **percentile of the lowest mean squared difference (MSD) between simulated δ^13^C-CH_4_ values and observations.** The runs were grouped by bottom-up estimates with different colors for major CH_4_ source categories. The number of runs grouped by different CH_4_ source categories is listed in the legend.

**Fig. S4. Hovmöller diagrams of the change rate of zonal annual CH_4_ emission anomalies for major CH_4_ sources (Table S1).** The annual change rate for equal latitudinal areas is calculated as the time derivative of the CH_4_ anomaly relative to its 2000 level. Note that the sine latitude is approximately equal to the latitudes at the right axis (sin. lat of 0.75 roughly equal to Lat. 45°N)*.* IFF, AGW, WET, and BB represent industrial fossil fuel, agricultural and waste, wetlands, and biomass burning sectors, respectively. The CH_4_ source estimates for IFF and AGW are from the inventories EDGAR4.2, EDGAR.4.3.2, GAINS, SCH150, and WOLF2017. REN and CRU are two wetland CH_4_ estimates from wetland model LPJ-wsl, whereas GFED is a bottom-up biomass burning dataset.

**Fig. S5. Latitudinal gradients (north: positive, south: negative) of weighted average wetland δ^13^C-CH_4_** **values.** The 1000 spatial maps were calculated using Monte Carlo techniques following a normal distribution.


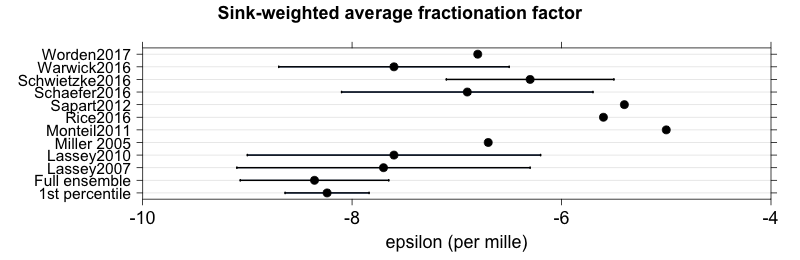


**Fig. S6. Comparison of the mean fractionation factor ε in the sink processes between this study and literature studies.** The mean fractionation factor and 1 sigma range for the full ensemble and most likely scenarios (1^st^ percentile of lowest MSD) are calculated by fitting with specific emission scenarios using parameter estimation. For Warwick2016, which does not explicitly show ε, the sink-weighted average fractionation factor was calculated based on individual ε_sink_ values from Lassey2007, where the ε_sink_ values of OH oxidation in the troposphere, soil removal, stratospheric loss and Cl sink are set to -4.65‰, -20‰, -3‰, and -60‰, respectively.

**Fig. S7. Same as Fig. 5 (bottom) but including a bar plot calculated using the full ensemble (top, N=96,000) of the box model simulations.**

**
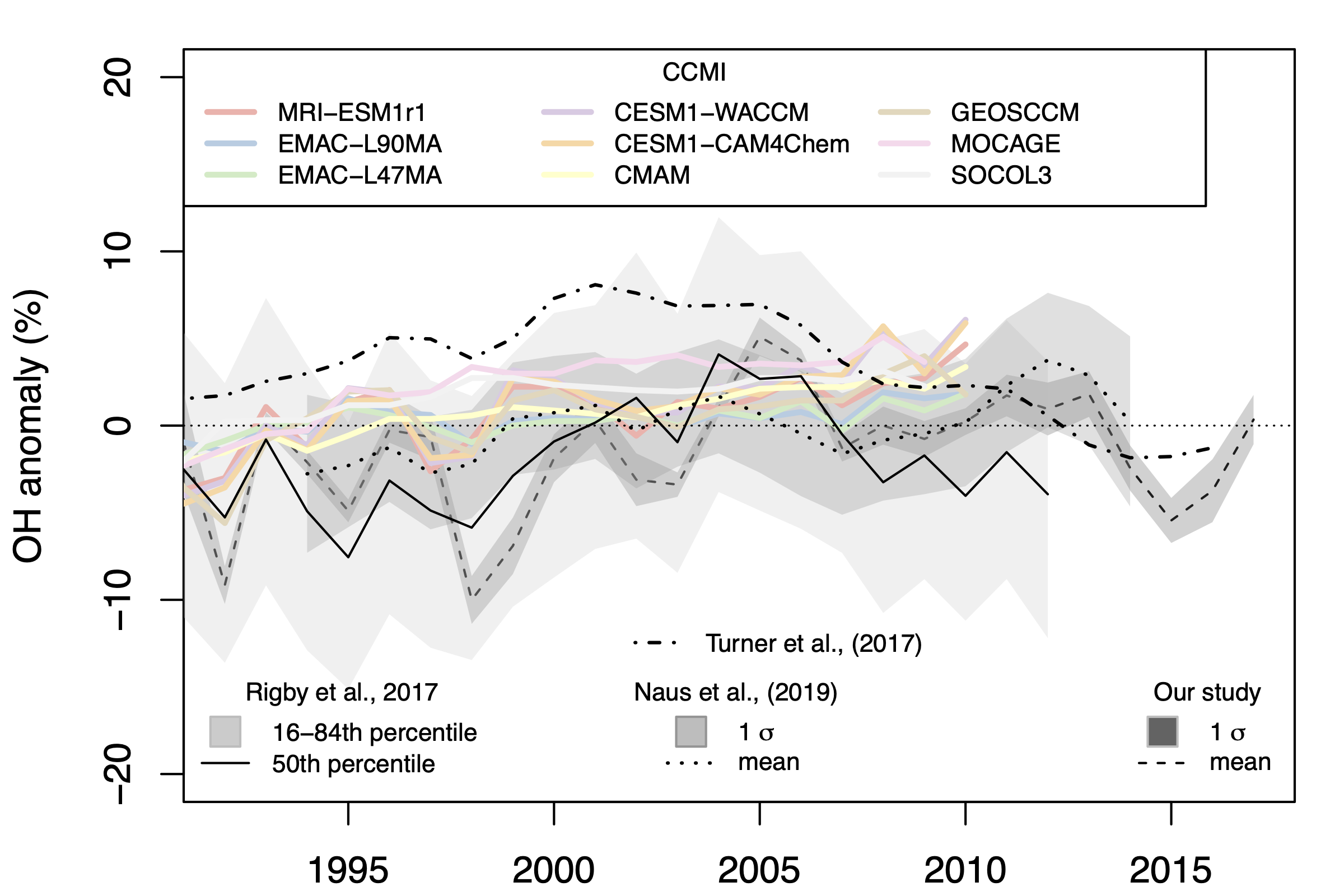
**

**Fig. S8. Same as Fig. S1 but for the OH time series of the most likely scenarios (N=960) that are within the 1^st^ percentile of lowest MSD.**


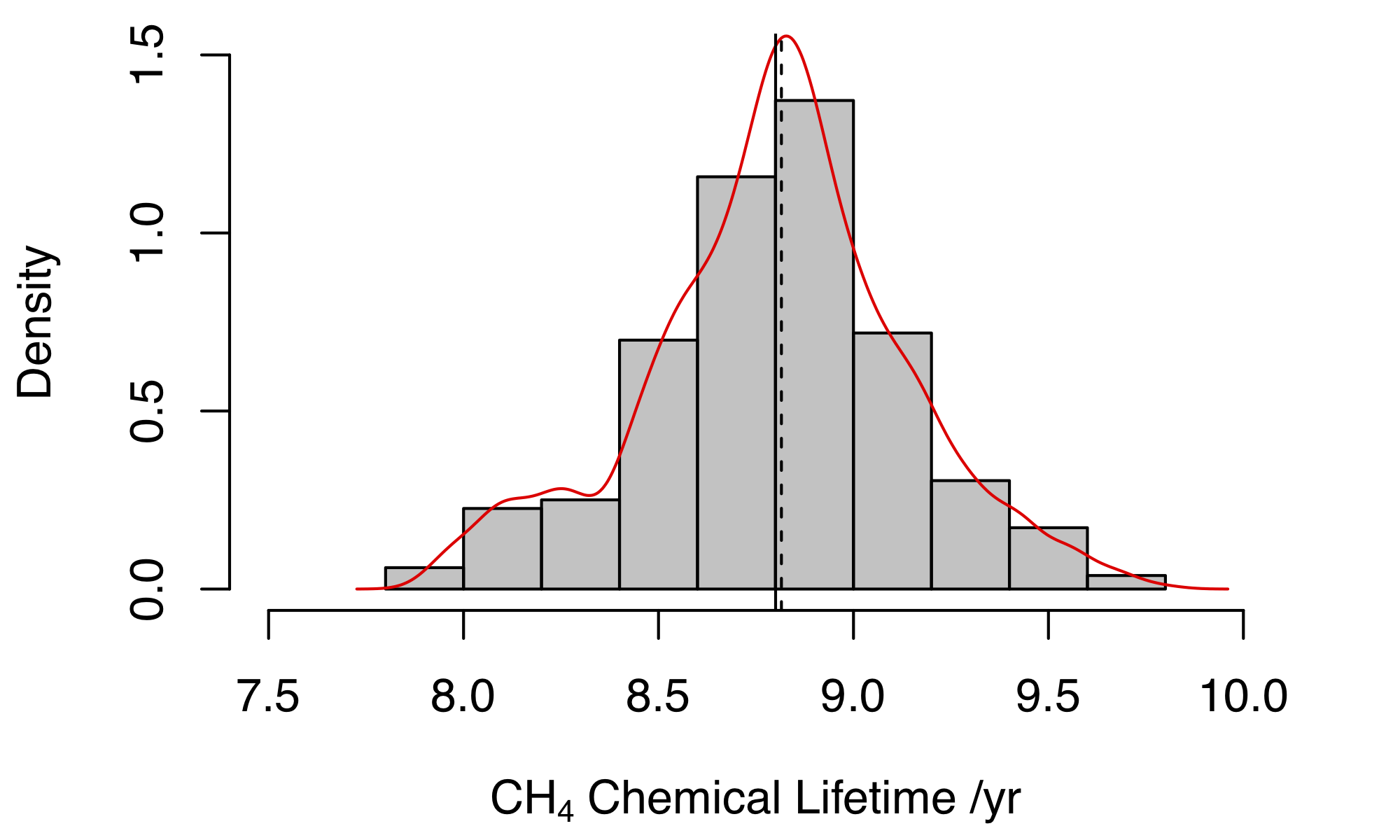


**Fig. S9. Probability distribution for the global tropospheric chemical lifetime of CH_4_ over 96,000 box model runs.** Solid and dashed lines are the mean and median, respectively.

**Fig. S10. Time series of emission-weighted δ^13^C-CH_4_ signatures for biomass burning using GFED and Worden2017 estimates.** NH and SH represent the Northern Hemisphere and Southern Hemisphere, respectively. ‘Old’ and ‘New’ represent the emission-weighted signature using -12.5‰ and -17.9‰ for C4 biomass burning, respectively.


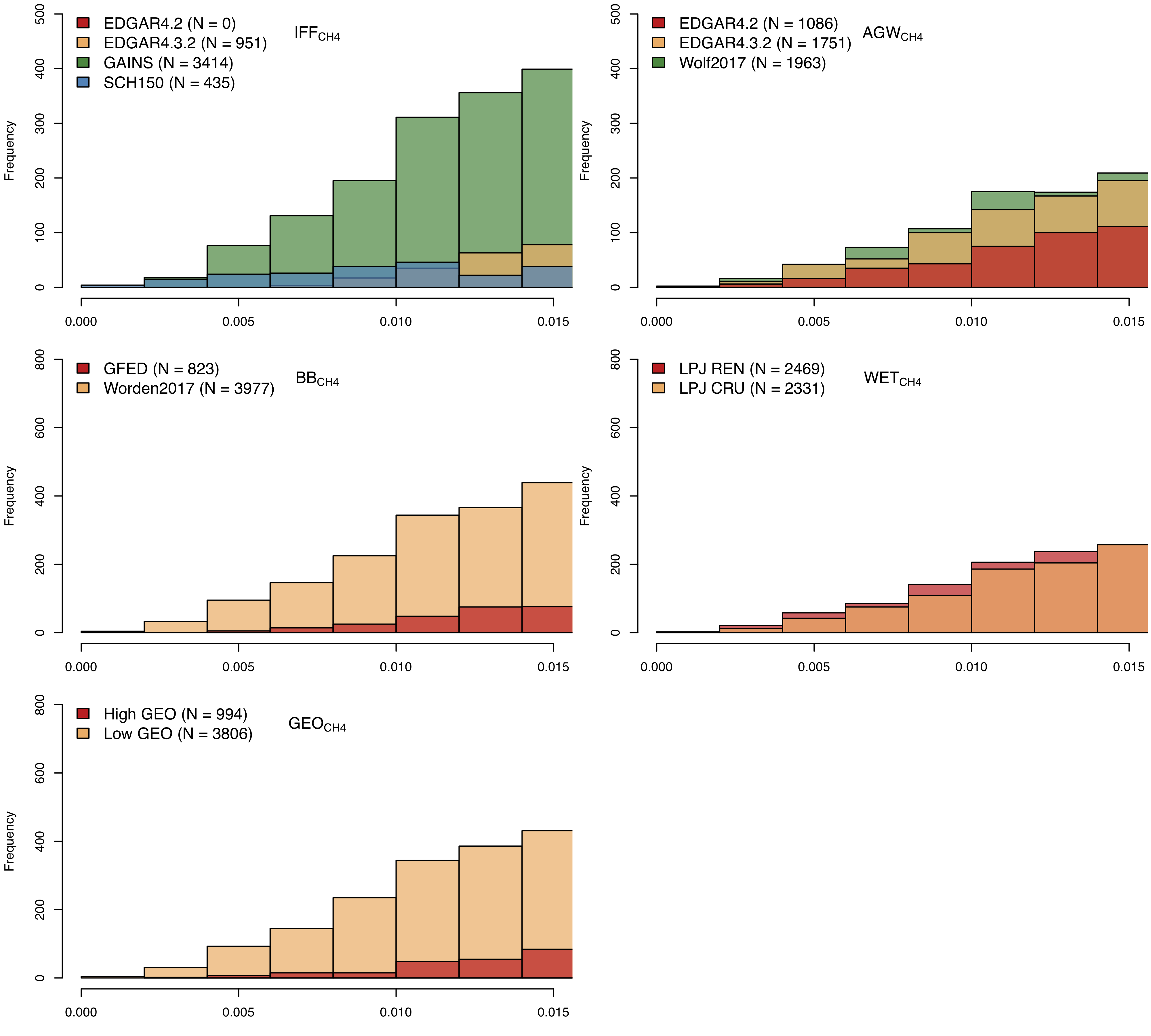


**Fig. S11. Same as Fig. S3 but using a threshold of 5^th^ percentile.**


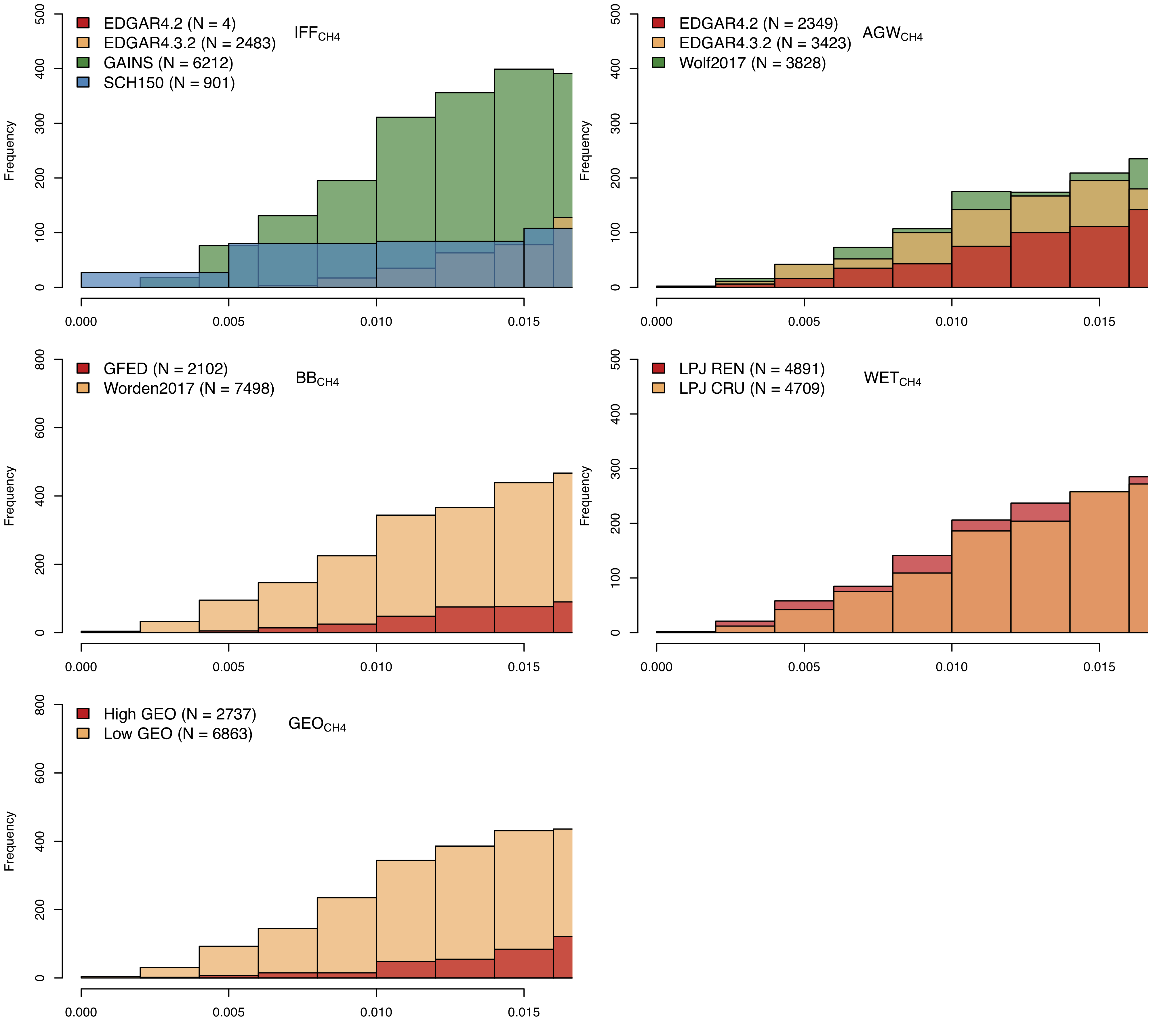


**Fig. S12. (Continued) Same as Fig. S3 but using a threshold of 10^th^ percentile.**

**Tables**

**Table S1 | List of data sources for CH_4_ emissions and sinks.**

| Category | Sub-Category | Source Name | Time period | Resolution | Isotopic Calculation | Time step |
| --- | --- | --- | --- | --- | --- | --- |
| IFF_CH4_ | Coal, Natural Gas/Oil | EDGARv4.2^a^ | 1970-2012 | 0.1° | by grid | Annual |
|  |  | EDGARv4.3.2 | 1970-2012 | 0.1° | by grid | Annual |
|  |  | GAINS | 1980-2012 | 0.5 | by grid | Annual |
|  |  | SCH150 | 1980-2011 | 1° | by grid | Annual |
| AGW_CH4_ | Livestock, Rice, Landfill/Waste | EDGARv4.2^a^ | 1970-2012 | 0.1° | by grid | Annual |
|  |  | EDGARv4.3.2 | 1970-2012 | 0.1° | by grid | Annual |
|  |  | WOLF2017^b^ | 2000-2013 | 0.05° | by grid | Annual |
| BB_CH4_ | N/A | GFED | 1980-2017 | 0.25° | by grid | Monthly |
|  |  | Worden2017 | 2001-2013 | 4°×5° | by grid | Annual |
| WET_CH4_ | N/A | LPJ with CRU | 1980-2017 | 0.5° | by grid | Monthly |
|  |  | LPJ with Reanalyses | 1980-2017 | 0.5° | by grid | Monthly |
| GEO_CH4_ | N/A | Etiope et al., (2018) | Climatology | 1° | by grid | Constant |
|  |  | Petreko et al., (2017) | Constant | Single Value | N/A | N/A |
| Soil Sink | N/A | Murguia-Flores et al.,  (2017) | Climatology | 0.25° | NA | Constant |

Notes: ^a^ EDGARv.4.2 represents a harmonized dataset merging EDGARv4.2 that covers 1970-2008 and EDGARv4.2FT2010 and EDGARv4.2FT2012 that extends the time series to 2012. ^b^WOLF2017 represents the sum of livestock CH_4_ emissions from Wolf et al. (2017) and rice and landfill/waste emissions from EDGAR 4.3.2.

**Table S2| Statistics of key variables (Unit: head) for major CH_4_ sources.** The Coal production is from IEA database (<https://www.iea.org/data-and-statistics/charts/world-total-coal-production-1971-2020>, Last Access Sep. 23th 2019). The average of 2000-2006 and 2007-2017 are derived from FAOSTAT database (<http://www.fao.org/faostat>, Last Access Sep. 23th 2019). The world urban population is from UNFCCC (2021).

| Year | Coal production (Mt) | Livestock stock (10^3^ head) | Urban Population (billion) |
| --- | --- | --- | --- |
| 1993 | 4327 | 3,219,548 | 2.442 |
| 1994 | 4402 | 3,252,508 | 2.499 |
| 1995 | 4557 | 3,261,377 | 2.559 |
| 1996 | 4622 | 3,283,479 | 2.617 |
| 1997 | 4621 | 3,230,847 | 2.675 |
| 1998 | 4569 | 3,260,497 | 2.734 |
| 1999 | 4499 | 3,295,301 | 2.794 |
| 2000 | 4638 | 3,325,152 | 2.854 |
| 2001 | 4860 | 3,319,481 | 2.919 |
| 2002 | 4914 | 3,339,462 | 2.988 |
| 2003 | 5263 | 3,380,498 | 3.057 |
| 2004 | 5663 | 3,451,552 | 3.128 |
| 2005 | 6022 | 3,526,145 | 3.201 |
| 2006 | 6367 | 3,553,065 | 3.274 |
| 2007 | 6644 | 3,608,346 | 3.346 |
| 2008 | 6842 | 3,630,842 | 3.422 |
| 2009 | 6968 | 3,642,222 | 3.498 |
| 2010 | 7354 | 3,644,759 | 3.475 |
| 2011 | 7833 | 3,683,766 | 3.645 |
| 2012 | 7936 | 3,731,191 | 3.724 |
| 2013 | 7976 | 3,779,367 | 3.800 |
| 2014 | 7939 | 3,796,950 | 3.877 |
| 2015 | 7699 | 3,863,463 | 3.956 |
| 2016 | 7293 | 3,924,322 | 4.036 |
| 2017 | 7545 | 3,962,268 | 4.116 |
|  |  |  |  |

Table S3| Correlation matrix for Coal, Livestock, and Waste CH_4_ emissions with corresponding inventory statistics from Table S2.

| Dataset | Coal production | Livestock stock | Urban population |
| --- | --- | --- | --- |
| EDGAR4.2 | 0.994 | 0.982 | 0.971 |
| EDGAR4.3.2 | 0.988 | 0.991 | 0.999 |
| GAINS | 0.985 | N/A | N/A |
| SCH150 | 0.997 | N/A | N/A |
| Wolf2017 | N/A | 0.961 | N/A |

**Table S4 | Unweighted global mean in isotopic signature and corresponding uncertainty (1-σ) used in the Monte Carlo estimation for source categories.**

| Source type | δ^13^C-CH_4_ (‰) | Reference |
| --- | --- | --- |
| Coal^*^ | -43.7±3.8 | [29,31] |
| Natural Gas & Oil^*^ | -44.7±4.5 | [29,31] |
| Livestock^*^ | -65.5±3.3 | [29,31] |
| Rice | -62.2±3.8 | [29] |
| Landfill/Waste | -56.0±7.1 | [29] |
| Biomass Burning^*^ | -24.6±2.2 | [29,31] |
| Wetlands^*^ | -61.6±5.3 | [32] |
| Geological Source | -42.5±7.0 | [29] |
| Termites | -63.4±5.7 | [29] |
| Freshwater | -61.5±5.0 | [29] |
| Wild Animal | -65.4±2.8 | [29] |
| Others | -44.8±2.0 | [29] |

*Mean value of the spatial distribution

**SI References**

1. Schaefer H, Fletcher SEM, Veidt C *et al.* A 21st-century shift from fossil-fuel to biogenic methane emissions indicated by ^13^CH_4_. *Science* 2016;**352**:80–4.

2. Turner AJ, Frankenberg C, Wennberg PO *et al.* Ambiguity in the causes for decadal trends in atmospheric methane and hydroxyl. *Proceedings of the National Academy of Sciences* 2017;**114**:5367–72.

3. Houweling S, van der Werf GR, Klein Goldewijk K *et al.* Early anthropogenic CH_4_ emissions and the variation of CH_4_ and ^13^CH_4_ over the last millennium. *Global Biogeochemical Cycles* 2008;**22**, DOI: 10.1029/2007GB002961.

4. Schwietzke S, Sherwood OA, Bruhwiler LMP *et al.* Upward revision of global fossil fuel methane emissions based on isotope database. *Nature* 2016;**538**:88–91.

5. Schwietzke S, Griffin WM, Matthews HS *et al.* Global Bottom-Up Fossil Fuel Fugitive Methane and Ethane Emissions Inventory for Atmospheric Modeling. *ACS Sustainable Chemistry & Engineering* 2014;**2**:1992–2001.

6. Höglund-Isaksson L. Bottom-up simulations of methane and ethane emissions from global oil and gas systems 1980 to 2012. *Environmental Research Letters* 2017;**12**:024007.

7. Wolf J, Asrar GR, West TO. Revised methane emissions factors and spatially distributed annual carbon fluxes for global livestock. *Carbon Balance and Management* 2017;**12**:16.

8. Marthews TR, Dadson SJ, Lehner B *et al.* High-resolution global topographic index values for use in large-scale hydrological modelling. *Hydrology and Earth System Sciences* 2015;**19**:91–104.

9. Zhang Z, Zimmermann NE, Kaplan JO *et al.* Modeling spatiotemporal dynamics of global wetlands: comprehensive evaluation of a new sub-grid TOPMODEL parameterization and uncertainties. *Biogeosciences* 2016;**13**:1387–408.

10. Zhang Z, Zimmermann NE, Stenke A *et al.* Emerging role of wetland methane emissions in driving 21st century climate change. *Proceedings of the National Academy of Sciences* 2017;**114**:9647–52.

11. Zhang Z, Zimmermann NE, Calle L *et al.* Enhanced response of global wetland methane emissions to the 2015–2016 El Niño-Southern Oscillation event. *Environmental Research Letters* 2018;**13**:074009.

12. Lunt MF, Palmer PI, Feng L *et al.* An increase in methane emissions from tropical Africa between 2010 and 2016 inferred from satellite data. *Atmospheric Chemistry and Physics* 2019;**19**:14721–40.

13. Yin Y, Chevallier F, Ciais P *et al.* Accelerating methane growth rate from 2010 to 2017: leading contributions from the tropics and East Asia. *Atmospheric Chemistry and Physics* 2021;**21**:12631–47.

14. Zhang Y, Jacob DJ, Lu X *et al.* Attribution of the accelerating increase in atmospheric methane during 2010–2018 by inverse analysis of GOSAT observations. *Atmospheric Chemistry and Physics* 2021;**21**:3643–66.

15. Werf GR van der, Randerson JT, Giglio L *et al.* Global fire emissions estimates during 1997–2016. *Earth System Science Data* 2017;**9**:697–720.

16. Worden JR, Bloom AA, Pandey S *et al.* Reduced biomass burning emissions reconcile conflicting estimates of the post-2006 atmospheric methane budget. *Nature Communications* 2017;**8**:2227.

17. Petrenko VV, Smith AM, Schaefer H *et al.* Minimal geological methane emissions during the Younger Dryas–Preboreal abrupt warming event. *Nature* 2017;**548**:443–6.

18. Hmiel B, Petrenko VV, Dyonisius MN *et al.* Preindustrial ^14^CH_4_ indicates greater anthropogenic fossil CH4 emissions. *Nature* 2020;**578**:409–12.

19. Etiope G, Milkov A, Derbyshire E. Did geologic emissions of methane play any role in Quaternary climate change? *Global and Planetary Change* 2008;**61**:79–88.

20. Dalsøren SB, Myhre G, Hodnebrog Ø *et al.* Discrepancy between simulated and observed ethane and propane levels explained by underestimated fossil emissions. *Nature Geoscience* 2018;**11**:178–84.

21. Etiope G, Sherwood Lollar B. Abiotic methane on earth. *Reviews of Geophysics* 2013;**51**:276–99.

22. Etiope G, Ciotoli G, Schwietzke S *et al.* Gridded maps of geological methane emissions and their isotopic signature. *Earth Syst Sci Data* 2019;**11**:1–22.

23. Saunois M, Bousquet P, Poulter B *et al.* The global methane budget 2000–2012. *Earth System Science Data* 2016;**8**:697–751.

24. Saunois M, Stavert AR, Poulter B *et al.* The Global Methane Budget 2000–2017. *Earth System Science Data* 2020;**12**:1561–623.

25. Allen GH, Pavelsky TM. Global extent of rivers and streams. *Science* 2018;**361**:585–8.

26. Messager ML, Lehner B, Grill G *et al.* Estimating the volume and age of water stored in global lakes using a geo-statistical approach. *Nature Communications* 2016;**7**:13603.

27. Zhang Z, Fluet-Chouinard E, Jensen K *et al.* Development of the global dataset of Wetland Area and Dynamics for Methane Modeling (WAD2M). *Earth System Science Data* 2021;**13**:2001–23.

28. Saunois M, Bousquet P, Poulter B *et al.* The Global Methane Budget 2000–2012. *Earth System Science Data* 2016;**8**:697–751.

29. Sherwood OA, Schwietzke S, Arling VA *et al.* Global Inventory of Gas Geochemistry Data from Fossil Fuel, Microbial and Burning Sources, version 2017. *Earth System Science Data* 2017;**9**:639–56.

30. Still CJ, Berry JA, Collatz GJ *et al.* Global distribution of C3 and C4 vegetation: Carbon cycle implications. *Global Biogeochemical Cycles* 2003;**17**:6-1-6–14.

31. Feinberg AI, Coulon A, Stenke A *et al.* Isotopic source signatures: Impact of regional variability on the δ13CH4 trend and spatial distribution. *Atmospheric Environment* 2018;**174**:99–111.

32. Ganesan AL, Stell AC, Gedney N *et al.* Spatially Resolved Isotopic Source Signatures of Wetland Methane Emissions. *Geophysical Research Letters* 2018;**45**:3737–45.

33. Brownlow R, Lowry D, Fisher RE *et al.* Isotopic Ratios of Tropical Methane Emissions by Atmospheric Measurement: Tropical Methane δ ^13^ C Source Signatures. *Global Biogeochemical Cycles* 2017;**31**:1408–19.

34. Walter KM, Chanton JP, Chapin FS *et al.* Methane production and bubble emissions from arctic lakes: Isotopic implications for source pathways and ages. *Journal of Geophysical Research* 2008;**113**:G00A08.

35. McCalley CK, Woodcroft BJ, Hodgkins SB *et al.* Methane dynamics regulated by microbial community response to permafrost thaw. *Nature* 2014;**514**:478–81.

36. Poulter B, Bousquet P, Canadell JG *et al.* Global wetland contribution to 2000–2012 atmospheric methane growth rate dynamics. *Environmental Research Letters* 2017;**12**:094013.

37. Taylor KE. Summarizing multiple aspects of model performance in a single diagram. *Journal of Geophysical Research: Atmospheres* 2001;**106**:7183–92.

38. Rigby M, Montzka SA, Prinn RG *et al.* Role of atmospheric oxidation in recent methane growth. *Proceedings of the National Academy of Sciences* 2017;**114**:5373–7.

39. Naus S, Montzka SA, Pandey S *et al.* Constraints and biases in a tropospheric two-box model of OH. *Atmospheric Chemistry and Physics* 2019;**19**:407–24.
